# Novel tetrahydroisoquinolines as DHFR and CDK2 inhibitors: synthesis, characterization, anticancer activity and antioxidant properties

**DOI:** 10.1186/s13065-024-01139-w

**Published:** 2024-02-16

**Authors:** Eman M. Sayed, Etify A. Bakhite, Reda Hassanien, Nasser Farhan, Hanan F. Aly, Salma G. Morsy, Nivin A. Hassan

**Affiliations:** 1 Department of Chemistry, Faculty of Science, New Valley University, El-Kharja, 72511 Egypt; 2 Department of Chemistry, Faculty of Science, Assiut University, Assiut, 71516 Egypt; 3 Department of Therapeutic Chemistry, National Research Centre, El-Behooth St., Dokki, Cairo, 12622 Egypt; 4 Department of Cancer Biology, Cancer Immunology and Virology Unit, South Egypt Cancer Institute, Assiut University, Assiut, Egypt; 5 Department Cancer Biology, Pharmacology and Experimental Oncology Unit, South Egypt Cancer Institute, Assiut University, Assiut, Egypt

**Keywords:** Anticancers, Apoptosis, Cell cycle arrest, CDK2 inhibitor, DHFR inhibitor, Antioxidants, Tetrahydroisoquinolines, Tetrahydrothieno[2,3-*c*]isoquinolines

## Abstract

In this study, we synthesized new 5,6,7,8-tetrahydroisoquinolines and 6,7,8,9-tetrahydrothieno[2,3-*c*]isoquinolines based on 4-(*N*,*N*-dimethylamino)phenyl moiety as expected anticancer and/or antioxidant agents. The structure of all synthesized compounds were confirmed by spectral date (FT-IR, ^1^H NMR, ^13^C NMR) and elemental analysis. We evaluated the anticancer activity of these compounds toward two cell lines: A459 cell line (lung cancer cells) and MCF7 cell line (breast cancer cells). All tested compounds showed moderate to strong anti-cancer activity towards the two cell lines. Compound **7e** exhibited the most potent cytotoxic activity against A549 cell line (IC_50_: 0.155 µM) while compound **8d** showed the most potent one against MCF7 cell line (IC_50_: 0.170 µM) in comparison with doxorubicin. In addition, we examined the effect of compounds **7e** and **8d** regarding the growth of A549 and MCF7 cell lines, employing flow cytometry and Annexin V-FITC apoptotic assay. Our results showed that compound **7e** caused cell cycle arrest at the G2/M phase with a 79-fold increase in apoptosis of A459 cell line. Moreover, compound **8d** caused cell cycle arrest at the S phase with a 69-fold increase in apoptosis of MCF7 cell line. Furthermore, we studied the activity of these compounds as enzyme inhibitors against several enzymes. Our findings by docking and experimental studies that compound **7e** is a potent CDK2 inhibitor with IC_50_ of 0.149 µM, compared to the Roscovitine control drug with IC_50_ of 0.380 µM. We also found that compound **8d** is a significant DHFR inhibitor with an IC_50_ of 0.199 µM, compared to Methotrexate control drug with IC_50_ of 0.131 µM. Evaluation of the antioxidant properties of ten compounds was also studied in comparison with Vitamin C. Compounds **1**, **3**, **6**, **7c** and **8e** have higher antioxidant activity than Vitamin C which mean that these compounds can used as potent antioxidant drugs.

**Graphical Abstract:**

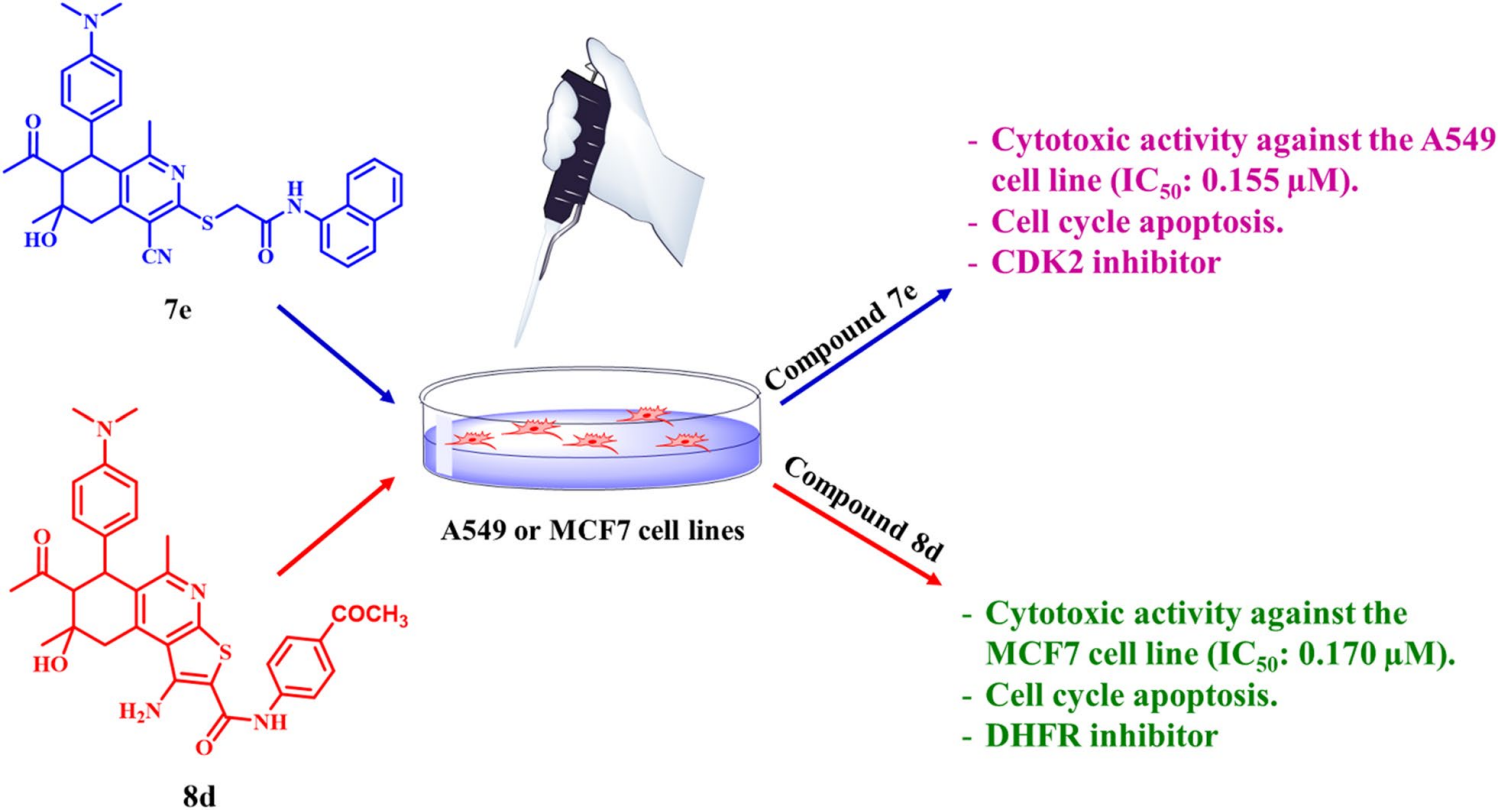

**Supplementary Information:**

The online version contains supplementary material available at 10.1186/s13065-024-01139-w.

## Introduction

Nowadays cancer is one of the most dangerous diseases in the world and it has risen to the position of the leading cause of death around the globed due to the inherent resistance of many types of cancer to conventional radiotherapy and chemotherapy [1]. So many strategies have been admitted treating cancer patients. One modality is through inhibition of cell cycle regulators enzymes of cancer cells such as inhibition of CDKs [2] and DHFR enzymes [3], epidermal growth factor (EGF) [2], Ras, and Tubulin proteins [4]. CDKs (cyclin-dependent kinases) are serine/threonine kinases enzymes that play a crucial role in regulating eukaryotic cell cycle [5], apoptosis, differentiation, and transcription. So, controlling CDKs activity has emerged as a promising therapeutic approach [5, 6]. CDK2 is one of CDK families which exist as an inactive form [5, 6], upon binding to its regulatory partners cyclin A or cyclin E. Which formed a functional heterodimeric complex to control cell cycle progression [7, 8]. Previous studies found that CDK2 is over-activated in many types of cancer [8]. Which makes CDK2 inhibitions is a desirable target for cancer treatment [9, 10]. CDK2 inhibitors could be classified as ATP-competitive and non-ATP-competitive based on their binding site [11]. Roscovitine and Flavopiridol are the most common commercial CDK2 inhibitors drugs where their structure based on heterocyclic moiety [12].

Dihydrofolate reductase enzyme (DHFR) is responsible for reduction of dihydrofolate (DHF) to tetrahydrofolate (THF). THF is essential for DNA synthesis, cell growth, and the production of raw materials for cell proliferation in both normal and cancer cells [13]. Therefor inhibitions of DHFR is an important target to prevent cell spreading [14]. Moreover DHFR enzyme required to maintain bacterial growth [15, 16]. Due to its critical role in nucleotide biosynthesis. Hence inhibitors of DHFR have been proven in as effective agents for treating bacterial infections [16]. Methotrexate is the most effective commercial drug for DHFR inhibition which contain heterocyclic atoms. In addition it has been approved to be effective in reducing cancer symptoms in children with acute lymphoblastic leukemia [14, 15].

Generally heterocyclic compounds were reported to be used as CDK2 inhibitors as reported in previous work such as pyridazines derivatives [5]. Oxindoles compounds [7], 6-Substituted 2-Arylaminopurines compounds [8], and Thiazolone compounds [11]. In addition, Recent literature showed that all new DHFR inhibitors contain heterocyclic moieties in their structure such as pyridine, quinoline and isoquinoline moieties [14, 17].

Isoquinoline ring is one of the heterocyclic compounds which reported to has various biological activities, including antimicrobial [18], anti-oxidant [19], anti-inflammatory [19, 20], antipyretic [20], antihypertensive [21], antitumor [22–25] and anti-proliferative effects [26, 27]. Many isoquinoline alkaloids, including cepharanthine, berberine, and tetrandrine, have shown anti-inflammatory effect [28]. Therefore, a huge effort has been spent in developing novel and effective isoquinoline derivatives. Furthermore, increased interest in partially hydrogenated isoquinoline derivatives is related to the presence of an isoquinoline fragment in molecules of many alkaloids, which give new biologically active compounds. Synthetic 1,2,3,4- and 5,6,7,8-tetrahydroisoquinoline derivatives were reported to exhibit antitumor [29–32], antihypertensive and neurotropic activities [33].

In view of the above observations, the current work was designed to synthesize and characterize some new (5,6,7,8-tetrahydroisoquinolin-3-yl)Thio compounds and related 6,7,8,9-tetrahyrothieno[2,3-*c*]isoquinolines incorporating 4-(*N*,*N*-dimethylamino) phenyl moiety to be examined as anticancer agents and antioxidant drugs. Dimethylamino moiety was chosen in this work because of its remarkable antioxidant activities [34] as they associate to the proton donors active groups in the surfaces like amino or methyl groups. These groups can interact by inter molecular reactions on the surface of DPPH to give antioxidant activities through hydrogen atom transfer reaction [35] in comparison with vitamin C drug. In addition to the tetrahydroisoquinolines anticancer [31, 32] properties in comparison with doxorubicin control and compounds **7e** and **8d** were the most potent compounds. Furthermore, the effect of compounds **7e** and **8d** on induced apoptosis and cell cycle arrest of the cancer cell lines were also included. Moreover, the enzyme inhibitory activities and molecular docking of two selective tetrahydroisoquinolines **7e** and **8d** were studied**.**

## Materials and methods

### Chemicals and instrumentations

Chemicals: chemicals of this work (4-(*N*,*N*-dimethylaminobenzaldhyde, Cyanothioacetamide, Piperidine, Methyl iodide, Ethyl Chloroacetate, 2-Chloroacetamide, Chloroacetonitrile or* N*-aryl-2-Chloroacetamides, Ethanol, Sodium acetate.3H_2_O, Sodium carbonate) were purchased from Sigma Aldrich Co.

Instrumentations: Melting points were determined on a Gallan-Kamp apparatus and are uncorrected. The purity of the compounds was ensured by TLC and the spectroscopic analysis.

IR spectra were recorded on a Shimadzu 470 IR-spectrophotometer (KBr; *ν*_max_ in cm^−1^). The ^1^H and ^13^C NMR spectra were recorded on Varian A5 500 MHz spectrometer using DMSO-*d*_*6*_ as a solvent and tetramethylsilane (TMS) as an internal reference. Coupling constants (*J* values) are given in Hertz (Hz). Elemental analyses were performed on a Perkin Elmer 2400 LS Series CHN/O analyzer.

Cell lines: The in vitro human breast cancerous cell line (MCF7), lung cancerous  cell lines (A549) and normal cell lines were purchased from Serum and Vaccine formulation in Cairo-Egypt.

Molecular docking: Molecular docking studies were performed in (I Mole Lab for bioinformatics, Cairo, Egypt).

Softwares: The biological data was analyzed and plot by Graphpad prism, Cell qust, ANOVA, Origin Lab, AutoDock Vina 1.1.2, Mestrenova and Excel software.

### 7-Acetyl-4-cyano-1,6-dimethyl-6-hydroxy-8-(4-*N,N*-dimethylaminophenyl)-5,6,7,8-tetrahydroisoquinoline-3(2*H*)-thione (1)

A mixture of 2,4-diacetyl-5-hydroxy-5-methyl-3-(4-(*N*,*N*-dimethylaminophenyl) cyclohexanone (3.3 g,10 mmol), 2-cyanothioacetamide (1.0 g,10 mmol) and piperidine (0.8 mL, 10 mmol) in ethanol (30 mL) was refluxed for 2 h. The yellow crystals that formed on cooling were collected, washed with methanol, and dried in air to give compound **1.** Yield: 98%; m. p: 283–284 °C. IR: 3432 (O–H), 3273 (N–H); 3142 (C–H, sp^2^); 2885 (C–H, sp^3^); 2216 (C≡N); 1709 (C=O); 1619 (C=N). ^1^H NMR: δ 13.78 (s, 1H, NH); 6.88 (d, 2H, *J* = 10 Hz, Ar–H); 6.61 (d, *J* = 10 Hz, 2H, Ar–H), 4.83 (s, 1H, OH); 4.27 (d, *J* = 10 Hz, 1H, C^8^H); 3.45 (d,* J* = 10 Hz, 2H: C^5^H and C^7^H), 3.28(s,1H, C^6^H) 2.87 (m, 7H: C^5^H and N(CH_3_)_2_); 2.09 (s, 3H, CH_3_, attached to C-1); 1.90 (s, 3H, COCH_3_); 1.24 (s, 3H, CH_3_) ppm. ^13^C NMR: δ 209.97, 182.75, 178.99, 174.94, 155.49, 155.41, 152.98, 149.21, 129.18, 129.03, 125.05, 116.90, 113.88, 113.01, 68.16, 68.07, 66.22, 56.49, 31.55, 28.11, 28.01, 19.01 ppm. Anal. Calcd. for C_22_H_25_N_3_O_2_S (395.17): C, 66.81; H, 6.37; N, 10.62%. Found: C, 66.61; H, 6.40; N, 10.78%.

### Reaction of compound 1 with methyl iodide, ethyl chloroacetate, 2-chloroacetamide, chloroacetonitrile or *N*-aryl-2-chloroacetamides 2a–e: synthesis of compounds 3, 4, 5, 6 and 7a–e

A mixture of **1** (3.95 g, 10 mmol), methyl iodide (0.7 mL, 10 mmol), ethyl chloroacetate (1 mL, 10 mmol), 2-chloroacetamide (0.93 g, 10 mmol), chloroacetonitrile (0.8 mL, 10 mmol)or *N-*aryl-2-chloroacetamide **2a–e** (10 mmol), and sodium acetate trihydrate (1.50 g, 11 mmol) in ethanol (100 mL) was refluxed for one hour. The reaction mixture was then allowed to stand at room temperature overnight. After that the precipitate was collected and recrystallized from ethanol as colorless crystals of title compounds **3, 4, 5, 6,** and **7a–e** respectively.

#### 7-Acetyl-4-cyano-1,6-dimethyl-3-methylthio-6-hydroxy-8-(4-*N*,*N*-dimethyl-aminophenyl)-5,6,7,8-tetrahydroisoquinoline (3)

Yield: 94%; m.p.: 162–163 °C. IR: 3510 (O–H); 2967, 2909 (C–H, sp^2^); 2217 (C≡N); 1696 (C=O, acetyl); 1612 (C=N). ^1^H NMR: δ 6.83 (d, *J* = 5 Hz, 2H, Ar–H); 6.61 (t, *J* = 5 Hz, 2H, Ar–H); 4.78 (s, 1H, OH), 4.39 (d, *J* = 5 Hz, 1H, C^8^H), 3.18 (dd,* J* = 7,10 Hz, 3H: C^7^H and C^5^H_2_), 2.86 (m, 9H: SCH_3_ and N(CH_3_)_2_), 2.11(d,* J* = 7 Hz, 3H, at C-1), 2.00 (s, 3H, CH_3_, COCH_3_), 1.25(d, *J* = 10 Hz, 3H, CH_3_) ppm. ^13^C NMR: δ 209.69, 165.72, 161.03, 157.43, 149.23, 148.72, 130.71, 130.08, 128.60, 115.30, 112.40, 104.17, 67.58, 66.31, 43.28, 42.06, 31.12, 27.61, 24.78, 23.73, 14.54. Anal. Calcd. for C_23_H_27_N_3_O_2_S (409.18): C, 67.45; H, 6.65; N, 10.26%. Found: C, 67,42; H: 6.58, N; 10.30%.

#### Ethyl 2-((7-Acetyl-4-cyano-1,6-dimethyl-6-hydroxy-8-(4-*N*,*N*-dimethylamino-phenyl)-5,6,7,8-tetrahydroisoquinolin-3-yl)thio)acetate (4)

Yield: 78%; m.p.: 159–160 °C. IR: 3506 (O–H); 2983, 2964, 2809 (C–H, sp^3^); 2215 (C≡N); 1740 (C=O, ester); 1695 (C=O, acetyl). ^1^H NMR: δ 6.81 (d, *J* = 10 Hz, 2H, Ar–H), 6.58 (d, *J* = 10 Hz, 2H, Ar–H), 4.81 (s, 1H, OH), 4.38 (d, *J* = 9 Hz, 1H, C^8^H), 4.05 (m, 4H: SCH_2_ and OCH_2_), C^5^H and), 3.22 (d,* J* = 10 Hz, 1H, C^5^H), 2.87 (d, *J* = 10 Hz, 8H: C^7^H, C^5^H and N(CH_3_)_2_), 2.11 (s, 3H, CH_3_, at C-1), 1.93 (s, 3H, COCH_3_), 1.25 (s, 3H, CH_3_), 1.12 (d, *J* = 5 Hz, 3H, CH_3_ of ester group) ppm. ^13^C NMR: δ 209.62, 168.58, 160.96, 156.15, 149.43, 148.74, 130.57, 128.63, 115.09, 112.38, 103.71, 67.59, 66.28, 60.90, 42.02, 40.00, 31.98, 31.10, 27.58, 24.49, 14.00. Anal. Calcd. for C_26_H_31_N_3_O_4_S(481.20): C, 64.84; H, 6.49; N, 8.72%. Found: C, 64.98; H, 6.44; N, 8.51%.

#### 2-[(7-Acetyl-4-cyano-1,6-dimethyl-6-hydroxy-8-(4-*N*,*N*-dimethylamino-phenyl)-5,6,7,8-tetrahydroisoquinolin-3-yl)thio]acetamide (5)

Yield: 85%; m.p.: 196–197 °C. IR: 3562 (O–H); 3436, 3295, 3181 (NH_2_); 2971, 2809 (C–H, sp^3^); 2219 (C≡N); 1698 (C=O, acetyl); 1667 (C=O, amide). ^1^H NMR: δ 7.50 (s, 1H, NH), 7.05 (s, 1H, NH), 6.82 (d, *J* = 10 Hz, 2H, Ar–H), 6.60 (d,* J* = 9 Hz, 2H, Ar–H), 4.75 (s, 1H, OH), 4.39 (d, *J* = 15 Hz, 1H, C^8^H), 3.88 (d,* J* = 12 Hz, 15 Hz, 2H, SCH_2_), C^5^H and), 3.26 (d, *J* = 10 Hz, 1H, C^5^H), 2.89 (m, 8H: C^7^H, C^5^H and N(CH_3_)_2_), 2.11 (s, 3H, CH_3_, at C-1), 1.99 (s, 3H, COCH_3_), 1.26 (s, 3H, CH_3_) ppm. ^13^C NMR: δ 210.02, 169.55, 161.45, 157.33, 149.80, 149.20, 131.21, 130.84, 129.14, 115.73, 112.95, 104.19, 68.07, 66.77, 43.77, 42.50, 33.82, 31.59, 28.07, 25.11.

Anal. Calcd. for C_24_H_28_N_4_O_3_S (452.19): C, 63.69; H, 6.24; N, 12.38%. Found: C, 63.37; H, 6.18; N, 12.41%.

#### 2-[(7-Acetyl-4-cyano-1,6-dimethyl-6-hydroxy-8-(4-*N*,*N*-dimethylamino-phenyl)-5,6,7,8-tetrahydroisoquinolin-3-yl)thio]acetonitrile (6)

Yield:90%; m.p.: 145 °C. IR: 3537 (O–H); 2966, 2924,2801 (C–H, sp^3^); 2246 (C≡N, non conjugated); 2217 (C≡N, conjugated); 1698 (C=O, acetyl). ^1^H NMR: δ 6.85 (d, *J* = 10 Hz, 2H, Ar–H), 6.61 (d, *J* = 10 Hz 2H, Ar–H), 4.79 (s, 1H, OH), 4.44 (d, *J* = 8 Hz, 1H, C^8^H), 4.32 (s, 2H, SCH_2_), 3.27 (d, 1H, C^5^H), 2.92(d, *J* = 8 Hz, 2H, C^7^H and C^5^H), 2.89 (d, *J* = 10 Hz, 6H: N(CH_3_)_2_), 2.12 (s, 3H, CH_3_, at C-1), 2.07 (s, 3H, COCH_3_), 1.27 (s, 3H, CH_3_) ppm. ^13^C NMR: δ 210.26, 162.04, 154.32, 150.40, 149.25, 132.05, 130.91, 129.19, 118.20, 115.25, 112.95, 104.66, 68.09, 66.69, 43.83, 42.55, 31.63, 27.98, 25.14, 15.74 ppm. Anal. Calcd. for C_24_H_26_N_4_O_2_S (434.18): C, 66.33; H, 6.03; N, 12.89%. Found: C, 65.72; H, 5.71; N, 13.09%.

#### 2-[(7-Acetyl-4-cyano-1,6-dimethyl-6-hydroxy-8-(4-*N*,*N*-dimethylamino-phenyl)-5,6,7,8-tetrahydroisoquinolin-3-yl)thio]-*N*-phenylacetamide (7a)

Yield: 80%; m.p.: 209–210 °C. IR: 3459 (O–H); 3247 (N–H); 2971, 2805 (C–H, sp^3^); 2211 (C≡N); 1706 (C=O, acetyl); 1683 (C=O, amide). ^1^H NMR: δ 10.21 (s, 1H, NH), 7.52 (d, *J* = 10 Hz, 2H, Ar–H), 7.27 (t, *J* = 10 Hz, 2H, Ar–H), 7.02 (m, 1H, Ar–H), 6.80 (d, *J* = 10 Hz, 2H, Ar–H), 6.57 (d, *J* = 10 Hz, 2H, Ar–H), 4.80 (s, 1H, OH), 4.37 (d, *J* = 10 Hz, 1H, C^8^H), 4.1 (dd, *J* = 10 Hz, 13 Hz, 2H, SCH_2_), 3.23(d, *J* = 17 Hz, 1H, C^5^H), 2.87 (m, 4H: C^7^H and C^5^H), 2.83 (s, 6H, N(CH_3_)_2_), 2.10 (s, 3H, CH_3_, at C-1), 1.92 (s, 3H, COCH_3_), 1.24 (s, 3H, CH_3_). ^13^C NMR: δ 217.44, 209.63, 166.10, 160.95, 156.74, 149.35, 148.72, 138.90, 130.61, 130.48, 128,69, 128.61, 123.26, 119.04, 115.19, 112.38, 103.66, 67.57, 66.28, 43.29, 41.99, 34.68, 31.06, 27.58, 24.54. Anal. Calcd. for C_30_H_32_N_4_O_3_S (528.22): C, 68.16; H, 6.10; N, 10.60%. Found: C, 68.10; H, 6.15; N, 10.46%.

#### 2-[(7-Acetyl-4-cyano-1,6-dimethyl-6-hydroxy-8-(4-*N*,*N*-dimethylamino-phenyl)-5,6,7,8-tetrahydroisoquinolin-3-yl)thio]-*N*-(4-tolyl)acetamide (7b)

Yield: 95%; m.p.:198–199 °C. IR: 3436 (O–H); 3251 (N–H); 3119 (C–H, sp^2^); 2964,2908 (C–H, sp^3^); 2216 (C≡N); 1706 (C=O, acetyl); 1675 (C=O, amide). ^1^H NMR: δ 10.11 (s, 1H, NH), 7.39 (d, *J* = 9 Hz, 2H, Ar–H), 7.07 (d, *J* = 8 Hz, 2H, Ar–H), 6.80 (d, *J* = 9 Hz, 2H, Ar–H), 6.57 (d, *J* = 9 Hz, 2H, Ar–H), 4.79 (s, 1H, OH), 4.37 (d, *J* = 10 Hz, 1H, C^8^H), 4.085 (dd, *J* = 4, 7 Hz, 2H, SCH_2_), 3.23 (d, *J* = 17 Hz, 1H, C^5^H), 2.87 (m, 2H, C^7^H and C^5^H), 2.83 (s, 6H, N(CH_3_)_2_), 2.22 (s, 3H, CH_3_ of 4-tolyl group), 2.10 (s, 3H, CH_3_, at C-1), 1.92 (s, 3H, COCH_3_), 1.24 (s, 3H, CH_3_) ppm. ^13^C NMR: δ 209.62, 165.83, 160.93, 156.77, 149.33, 148.70, 136.40, 132.16, 130.61,130.45, 129.06, 128.61, 119.05, 115.18, 112.37, 103.64, 67.56, 66.27, 43.28, 41.98, 34.64, 31.05, 27.57, 24.53, 20.38 ppm. Anal. Calcd. For C_31_H_34_N_4_O_3_S (542.24): C, 68.61; H, 6.31; N, 10.32%. Found: C, 68.52; H, 6.45; N, 10.11%.

#### 2-[(7-Acetyl-4-cyano-1,6-dimethyl-6-hydroxy-8-(4-*N,N-*dimethylamino-phenyl)-,5,6,7,8-tetrahydroisoquinolin-3-yl)thio]-*N*-(4-chlorophenyl)acetamide (7c)

Yield: 96%; m.p.: 214–215 °C. IR: 3458 (O–H); 3242 (N–H); 2966, 2804 (C–H, sp^3^); 2214 (C≡N); 1685 (2C=O, acetyl and amide); 1610 (C=N). ^1^H NMR: δ 10.36 (s, 1H, NH), 7.55 (d, *J* = 10 Hz, 2H, Ar–H), 7.32 (t, *J* = 10 Hz, 2H, Ar–H), 6.80 (d, *J* = 9 Hz, 2H, Ar–H), 6.57 (d, *J* = 8 Hz, 2H, Ar–H), 4.80 (s, 1H, OH), 4.37 (d, *J* = 10 Hz, 1H, C^8^H), 4.11 (dd, *J* = 12,15 Hz 2H, SCH_2_), 3.23 (d, *J* = 17 Hz, 1H, C^5^H), 2.89 (m, 2H, C^7^H and C^5^H), 2.84 (s, 6H, N(CH_3_)_2_), 2.10 (s, 3H, CH_3_, at C-1), 1.90 (s, 3H, COCH_3_), 1.25 (s, 3H, CH_3_) ppm. ^13^C NMR: δ 209.62, 166.32, 160.93, 156.67, 149.34, 148.71, 137.86, 130.58, 130.49,128.60, 126.81, 120.56, 115.16, 112.36, 103.66, 67.51, 66.26, 43.28, 41.99, 34.69, 31.07, 27.57, 24.50 ppm. Anal. Calcd. For C_30_H_31_ClN_4_O_3_S (562.18): C, 63.99; H, 5.55; N, 9.95%. Found: C, 64.15; H, 5.48; N, 9.84%.

#### 2-[(7-Acetyl-4-cyano-1,6-dimethyl-6-hydroxy-8-(4-*N*,*N*-dimethylamino-phenyl)-5,6,7,8-tetrahydroisoquinolin-3-yl)thio]-*N*-(4-acetylphenyl)acetamide (7d)

Yield:93%; m.p.: 205 °C. IR: 3490 (O–H); 3244 (N–H); 3033 (C–H, sp^2^); 2922 (C–H, sp^3^); 2215 (C≡N); 1690 (3C=O, acetyl and amide); 1614 (C=N). ^1^H NMR: δ 10.62 (s, 1H, NH), 7.89 (d, *J* = 10 Hz, 2H, Ar–H), 7.67 (d, *J* = 10 Hz, 2H, Ar–H), 6.80 (d,* J* = 13 Hz, 2H, Ar–H), 6.55 (d, 2H, Ar–H), 4.80 (s, 1H, OH), 4.36 (d, *J* = 10 Hz, 1H, C^8^H), 4.15 (dd,* J* = 11, 13 Hz 2H, SCH_2_), 3.23 (d, *J* = 20 Hz, 1H, C^5^H), 2.87 (d, *J* = 12 Hz, 2H, C^7^H and C^5^H), 2.82 (s, 6H, N(CH_3_)_2_), 2.50 (s, 3H, COCH_3_ attached to phenyl group and overlapped with solvent proton), 2.10 (s, 3H, CH_3_, at C-1), 1.88 (s, 3H, COCH_3_), 1.24 (s, 3H, CH_3_). ^13^C NMR: δ 209.26, 196.36, 166.83, 160.75, 156.63, 149.38, 148.70,143.72, 131.68, 130.57, 129.45, 128.61, 118.22,115.33, 112.57, 103.72, 67.57, 66.27, 43.28, 41.97, 34.82, 31.05, 27.57, 26.34, 24.47. Anal. Calcd. for: C_32_H_34_N_4_O_4_S: (570.23): C, 67.35; H, 6.00; N, 9.82%. Found: C, 67.00; H, 5.88; N, 9.79%.

#### 2-[(7-Acetyl-4-cyano-1,6-dimethyl-6-hydroxy-8-(4-*N*,*N*-dimethylamino-phenyl)-5,6,7,8-tetrahydroisoquinolin-3-yl)thio]-*N*-(naphthalen-1-yl)acetamide (7e)

Yield: 88%; m.p.: 194–195 °C. IR: 3506 (O–H); 3288 (N–H); 3114 (C–H, sp^2^); 2968–2804 (C–H, sp^3^); 2217 (C≡N); 1696 (2 C=O, acetyl and amide); 1611 (C=N). ^1^H NMR: δ 10.20 (s, 1H, NH), 7.94 (d, *J* = 10 Hz, 2H, Ar–H), 7.75 (d, *J* = 7 Hz, 1H, Ar–H), 7.57 (d, *J* = 8 Hz, 1H, Ar–H), 7.46 (d, *J* = 10, 2H, Ar–H), 7.33 (m, 1H, Ar–H), 6.85 (d, *J* = 9 Hz, 2H, Ar–H), 6.59 (d, *J* = 8 Hz, 2H, Ar–H), 4.83 (s, 1H, OH), 4.42 (d, *J* = 10 Hz, 1H, C^8^H), 4.30 (dd, *J* = 15, 17 Hz, 2H, SCH_2_), 3.27 (d, *J* = 20 Hz, 1H, C^5^H), 2.93 (d, *J* = 10 Hz, 1H, C^7^H), 2.86 (m, 7H: C^5^H and N(CH_3_)_2_), 2.12 (s, 3H, CH_3_, at C-1), 2.04 (s, 3H, COCH_3_), 1.27 (s, 3H, CH_3_). ^13^C NMR: δ 202.84, 166.93, 161.10, 156.89, 149.39, 148.73, 133.61, 130.86, 128.69, 128.03, 125.48, 122.72, 121.71, 115.36, 112.24, 103.43, 67.61, 66.28, 43.33, 42.07, 34.17, 31.14, 27.60, 24.69. Anal. Calcd. for: C_34_H_34_N_4_O_3_S (578.24): C, 70.56; H, 5.92; N, 9.68%. Found: C, 70.43; H, 5.89; N, 9.85%.

### 7-Acetyl-1-amino-2-(*N*-arylcarbamoyl)-5,8-dimethyl-8-hydroxy-6-(4-*N*,*N*-dimethylaminophenyl)-6,7,8,9-tetrahydrothieno[2,3-*c*]isoquinolines 8a–d: general procedures

#### Method A

To a suspension of **7a–e** (10 mmol) in abs. ethanol (60 mL), anhydrous sodium carbonate (0.30 g) was added. The reaction mixture was refluxed for 3 h. The yellow crystals that formed while hot were collected, washed with water, dried in air, and then crystallized from dioxane to give **8a–e**.

##### 7-Acetyl-1-amino-5,8-dimethyl-8-hydroxy-6-(4-*N*,*N*-dimethylaminophenyl)-*N*-phenyl-6,7,8,9-tetrahydrothieno[2,3-c]isoquinoline-2-carboxamide (8a)

Yield: 96%; m.p.: 260 °C. IR:3501, 3451 (O–H, NH_2_ and NH); 3123 (C–H, sp^2^); 2990, 2810(C–H, sp^3^); 1695 (C=O, acetyl); 1631 (C=O, amide). ^1^H NMR: δ 9.40 (s, 1H, NH), 7.69 (d, *J* = 8 Hz, 2H, Ar–H), 7.33 (d, *J* = 8 Hz, 2H, Ar–H), 7.07 (m, 3H, Ar–H), 6.78 (br s, 2H, NH_2_), 6.59 (d, *J* = 9 Hz, 2H, Ar–H), 4.70 (br s, 1H, OH), 4.48 (d, *J* = 10 Hz, 1H, C^6^H), 3.57 (d, *J* = 17 Hz, 1H, C^9^H), 3.39 (d, 1H, C^7^H), 2.84 (m,7H: C^9^H and N(CH_3_)_2_), 2.14 (s, 3H, CH_3_, at C-5), 2.04 (s, 3H, COCH_3_), 1.29 (s, 3H, CH_3_). ^13^C NMR: δ 210.27, 164.37, 158.77, 155.97, 149.45, 148.61, 141.95, 138.89, 131.73, 130.04, 128.47, 128.34, 123.38, 122.88, 121.24, 112.43, 96.88, 67.18, 66.59, 42.39, 40.05, 31.19, 28.02, 24.65. Anal. Calcd. for C_30_H_32_N_4_O_3_S (528.22): C, 68.16; H, 6.10; N, 10.60%. Found: C, 68.02; H, 6.00; N, 10.27%.

##### 7-Acetyl-1-amino-5,8-dimethyl-8-hydroxy-6-(4-*N*,*N*-dimethylaminophenyl)-*N*-(4-tolyl)-6,7,8,9-tetrahydrothieno[2,3-*c*]isoquinoline-2-carboxamide (8b)

Yield:93%; m.p.: 289–290 °C. IR: 3394, 3327 (O–H, NH_2_, NH); 2915, 2798 (C–H, sp^3^); 1703 (C=O, acetyl); 1614 (C=N). ^1^H NMR: δ 9.33 (s, 1H, NH), 7.58 (s, 2H, Ar–H), 7.15 (s, 2H, Ar–H), 7.02 (d, *J* = 64 Hz, 2H, Ar–H), 6.76 (s, 2H, NH_2_), 6.59 (d, *J* = 10 Hz, 2H, Ar–H), 4.66 (s, 1H, OH), 4.48 (d, *J* = 94 Hz, 1H, C^6^H), 3.57 (m, 2H, C^9^H and C^7^H), 2.85 (m, 7H, C^9^H and N(CH_3_)_2_), 2.28 (s, 3H, CH_3_ of 4-tolyl group), 2.14 (s, 3H, CH_3_, at C-5), 2.03 (s, 3H, COCH_3_), 1.28 (s, 3H, CH_3_). ^13^C NMR: δ 209.98, 209.69, 164.24, 158.91, 158.61, 158.31, 158.14, 158.00, 155.72, 149.22, 142.52, 129.06, 123.34, 121,47, 121.42, 118.74, 118.28, 118.23, 118.19, 116.44, 114.14, 111.85, 103.52, 97.26, 67.27, 66,35, 43.94, 42.69, 31.03, 27.99, 24.46, 20.49 Anal. Calcd. for C_31_H_34_N_4_O_3_S (542.24) C, 68.61; H, 6.31; N, 10.32%. Found; C, 68.57; H, 6.66; N, 10.24%.

##### 7-Acetyl-1-amino-*N*-(4-chlorophenyl)-5,8-dimethyl-8-hydroxy-6-(4-*N*,*N*-dimethyl-aminophenyl)-6,7,8,9-tetrahydrothieno[2,3-*c*]isoquinoline-2-carboxamide (8c)

Yield: 83%; m.p.: 295 °C. IR: 3416, 3325 (O–H, NH_2_, NH); 2916 (C–H, sp^3^);1703 (C=O, acetyl); 1614 (C=N. ^1^H NMR: δ 9.67 (s, 1H, NH), 7.93 (s, 2H, NH_2_), 7.65 (d, *J* = 10 Hz, 2H, Ar–H), 7.35 (d, *J* = 10 Hz, 2H, Ar–H), 7.24 (d, *J* = 10 Hz, 2H, Ar–H), 7.06(d, *J* = 10 Hz, 2H, Ar–H), 4.66 (s, 1H, OH), 3.59 (d, *J* = 17 Hz, 1H, C^6^H), 3.31 (d, 1H, C^9^H), 3.03 (m, 7H: C^7^H and N(CH_3_)_2_), 2.83 (d, *J* = 10, 1H, C^9^H), 2.14 (s, 3H, CH_3_, at C-5), 2.02 (s, 3H, COCH_3_), 1.29(s, 3H, CH_3_). ^13^C NMR: δ 165.34, 161.46, 158.98, 158.69, 158.39, 158.09, 155.94, 153.55, 149.8, 147.95, 143.16,139.75, 138.01, 129.51, 128.73, 128.36, 127.23, 123.26, 122.78, 118.39, 116.64,114.33, 112.03, 96.85, 67.06, 66.14, 44.02, 42.27,42,21, 31.12, 28.00, 24.48. Anal. Calcd. for C_30_H_31_ClN_4_O_3_S (562.18): C, 63.99; H, 5.55; N, 9.95%. Found: C, 64.15; H, 5.49; N, 9.62%.

##### 7-Acetyl-*N*-(4-acetylphenyl)-1-amino-5,8-dimethyl-8-hydroxy-6-(4-*N*,*N*-dimethylaminophenyl)-6,7,8,9-tetrahydrothieno[2,3-*c*]isoquinoline-2-carboxamide (8d)

Yield:89%; m.p.: 301–302 °C. IR: 3424 (O–H); 3320 (N–H); 2916 (C–H, sp^3^); 1705 (C=O, acetyl); 1681 (C=O, amide). ^1^H NMR: δ 9.71 (s, 1H, NH), 7.91 (m, 6H, Ar–H), 7.17 (d, *J* = 10 Hz, 2H, Ar–H), 7.04 (s, 2H, NH_2_), 4.63 (s, 1H, OH), 3.60 (d, *J* = 8 Hz,1H, C^6^H), 3.39 (d, *J* = 10 Hz, 1H, C^9^H), 3.02 (s, 6H, N(CH_3_)_2_), 2.84 (d, *J* = 10 Hz, 1H, C^7^H), 2.53 (s, 4H: C^9^H and COCH_3_ attached to phenyl group and ovellaped with solvent protons), 2.17 (s, 3H, CH_3_, at C-5), 2.03 (s, 3H, COCH_3_), 1.30 (s, 3H, CH_3_). ^13^C NMR: δ 202.91, 196.84,164.62, 159.00, 158.78, 158.70, 158.40, 158.00, 156.21, 150.29,143.71, 142.98, 131.74, 129.63, 129.37, 129.13,128.98, 123.01, 120.02,118.96, 117.58, 116.64, 114.34, 112.03, 96.38, 67.33, 66.28, 43.46, 42.72, 42.20, 31.13, 28.00, 26.43, 24.57. Anal. Calcd. for: C_32_H_34_N_4_O_4_S: (570.23): C, 67.35; H, 6.00; N, 9.82%. Found: C, 67.51; H, 6.09; N, 9.74%.

##### 7-Acetyl-1-amino-*N-*(naphthalen-1-yl)-5,8-dimethyl-8-hydroxy-6-(4-*N*,*N*-dimethylminophenyl)-6,7,8,9-tetrahydrothieno[2,3-*c*]isoquinoline-2-carboxamide (8e)

Yield: 94%; m.p.: 288–290 °C. IR:3440, 3391 (O–H, NH_2_, NH); 3050 (C–H, sp^2^); 2910 (C–H, sp^3^); 1702 (C=O, acetyl); 1633 (C=O, amide). ^1^H NMR: δ 9.69 (s, 1H, NH), 7.51–7. 95 (m, 7H, Ar–H of 2-naphthyl group), 6.97 (br s, 2H, NH_2_), 6.78 (d,* J* = 15 Hz, 2H, Ar–H), 6.60 (d,* J* = 17 Hz, 2H, Ar–H), 4.65 (s, 1H, OH), 4.50 (d, *J* = 16 Hz,1H, C^6^H), 3.55 (d,* J* = 17 Hz, 1H, C^9^H), 3.38 (d,* J* = 13 Hz, 1H, C^7^H), 2.86 (d,* J* = 12 Hz, 7H: C^9^H and N(CH_3_)_2_), 2.14 (s, 3H, CH_3_, at C-5), 2.04 (s, 3H, COCH_3_), 1.28 (s, 3H, CH_3_). ^13^C NMR: δ 200.05, 165.03, 158.52, 156.05, 148.68, 141.88, 133.34, 131.80, 129.92, 128.47, 125.87, 123.46, 112.45, 67.23, 66.27, 42.63, 41.96, 31.4, 28.02, 24.63. Anal. Calcd for: C_34_H_34_N_4_O_3_S (578.24): C, 70.56; H, 5.92; N, 9.68%. Found: C, 70.79; H, 5.79; N, 9.42%.

#### Method B

A mixture of **1** (3.95 g, 10 mmol), *N-*aryl-2-chloroacetamide **2a–e** (10 mmol) and anhydrous sodium carbonate (1.35 g) in ethanol (100 mL) was refluxed for three hours. The precipitate that formed on cooling was collected and recrystallized from dioxane as yellow crystals of** 8a–e** (94–98%).

### Biological evaluation

#### In vitro cytotoxic activity

In vitro cytotoxic activity of all synthesized compounds against two human breast cell line (MCF7) and lung cell lines (A549) was evaluated according to the MTT method [23–25, 37, 38]. Firstly, Growth the cell line medium in 96 well tissue culture plate was injected with 10^5^ cells/mL (100 uL/plate well) of the cell line and incubated at 37 °C for 24 h to develop a monolayer sheet then the formed growth medium was poured from 96 well microtiter plates after the confluent sheet of cells. After that preparing the isoquinoline samples stock solutions in DMSO and diluted the concentrations to started from 0.0487, 0.0975, 0.195, 0.391, 0.781, 1.562, 3.125, 6.25, 12.50, 25.00 µM. Secondly, add 0.1 mL of each concentration tetrahydroisoquinoline sample to each plate. The plates were incubated at 37 °C. Thirdly MTT solution (5 mg/mL in PBS) is prepared. Add 20 µL of MTT solution to each well plates. And shaking in 150 rpm for 5 min, to mix the MTT into the media. Then incubate at (37 °C, 5% CO_2_) for 1–5 h. Finally read the optical density at 560 nm and subtract background at 620 nm.

#### Cell cycle analysis

The cell cycle arrests of compound **7e** against A549 and compound **8d** against MCF7 at their IC_50_ values were carried out according to Abcam method (code ab139418), (www.abcam.co.jp). Thus, A549 and MCF7 cells were collected and fixed with 75% ice-cold ethanol before being stored at − 20 °C for 1 h after being treated with an IC_50_ dose of our compounds **7e**, **8d**. Then centrifuged the cells and washed twice with ice-cold PBS, and incubated for 20 min at 4 °C. A cell cycle assay was used to assess the cell cycle (Propidium Iodide Flow Cytometry Kit [ab13941]. Then perform statistical analysis for the result by the Cell quest software on the cell fractions in sub-G0/G1, S, and G2/M phases [38].

#### Annexin-V FITC apoptosis assay

The Annexin-V FITC apoptosis assay of compounds **7e** against **A549** cell line and **8d** against **MCF7** cell line at their IC_50_ values were carried out according to (BioVision) protocol (code k101-25). (www.biovision.com). Thus, cell line were treated with the IC_50_ concentration of the compounds for 24 h then collected by trypsin, and centrifuged then rinsed with PBS and suspended in 0.5 mL of binding buffer, then dual-stained with Annexin V-FITC (5 μL) and propidium iodide (5 μL) in the dark for 15 min at RT. These stained cells were measured using flow cytometry with an excitation wavelength of 488 nm and an emission wavelength of 530 nm. The results were then analyzed with the Cell quest software [39–41].

#### Molecular docking

##### Protein preparation

The three-dimensional crystal structures of cyclin-dependent kinase 2 (CDK2, PDB ID 1AQ1) and dihydrofolate reductase (DHFR, PDB ID 1BOZ) were obtained from the Protein Data Bank (PDB). The protein structures were prepared using AutoDockTools 1.5.6. All water molecules were removed and hydrogen atoms were added. Gasteiger charges were assigned and nonpolar hydrogen were merged.

##### Ligand preparation

The 3D structures of ligand 1 (**7e** compound) and ligand 2 (**8d** compound) were built and energetically minimized using Avogadro 1.2.0 with the MMFF94 force field. Ligand atom types were assigned and rotatable bonds were defined using AutoDockTools. Both ligands were converted to PDBQT format required for docking calculations.

##### Molecular docking

Molecular docking studies were performed in **(I Mole Lab for bioinformatics, Cairo, Egypt)** by using AutoDock Vina 1.1.2. For each protein target, a docking grid box was generated to cover the active site based on a co-crystalized ligand. The exhaustiveness parameter was set to 8. Docking was performed with the prepared proteins and ligands to generate 9 binding poses per ligand. The best binding poses based on docking score were visually analyzed using Biovia Discovery Studio 2020 for interactions with key active site residues.

#### CDK2 inhibitors assay

The CDK2/cyclin A2 protein kinase assay was performed according to the bioscience protocol (code #79599) (www.bpsbioscience.com).

Firstly, prepare the master mixture (6 μL of 5 × Kinase assay buffer 1 + 1 µL of ATP (500 µM) + 5 µL of 10 × CDK substrate peptide 1 + 13 μL of distilled water).then add 25 μL of master mixture to every well of the 96-well plate. Add 20 ng of Cyclin A2 and 30 ng of different CDK2 mutant protein into the wells as indicated along with 0.155 µM of our synthesized compound **7e**. Incubate at 30 °C for 45 min. After the 45-min reaction, add 50 µL of Kinase-Glo Max reagent to each well. After that cover the plate with aluminum foil and incubate the plate for 15 min at RT. Then Measure luminescence after subtracted The value of blank from all readings using the microplate reader. The relative kinase activity of Cyclin A2/wild-type CDK2 group is set as 100%. The data was analysied and plot by Graphpad prism software [42, 43].

#### DHFR inhibitors assay

The DHFR inhibitors assay kit was performed according to abcam (code ab283374); (www.abcam.co.jp).

Firstly, Dilute 2 μL Dihydrofolate Reductase with798 μL DHFR Assay Buffer. Then add 98 μL of diluted Dihydrofolate Reductase into desired well(s) containing the out synthesized **8d** compound. Add 40 μL of diluted NADPH to each well containing the test samples. Incubate at room temperature for 10–15 min. Add 60 μL of diluted DHFR substrate to each well containing the test samples vortex briefly and keep on ice. Measure the absorbance immediately at 340 nm. Then calculate the inhibition concentration of **8d** compound. The data was analyzed and plot by Graphpad prism software [44, 45].

#### Antioxidant activity

The antioxidant activity of ten compounds was determined using DPPH [32–34]. A solution 1: prepared by dissolving DPPH (0.002 g) in ethanol (50 mL etnanol). Solution 2: prepared by dissolving different weights 0.05, 0.01 g of each sample in 1 mL of DMSO then take 10 µL of each sample solution with 1 mL ethanol. Then mix 1 mL of solution 1 with 1 mL of solution 2 then vortex the resulting mixture in the dark for about 30 min. The absorbance of the mixture was measured by spectrophotometer at λ_max_ = 517 nm against blank 1 mL absolute ethanol and compared to the ascorbic acid (Vitamin C).

## Results and discussion

### Synthesis

**Scheme 1:**
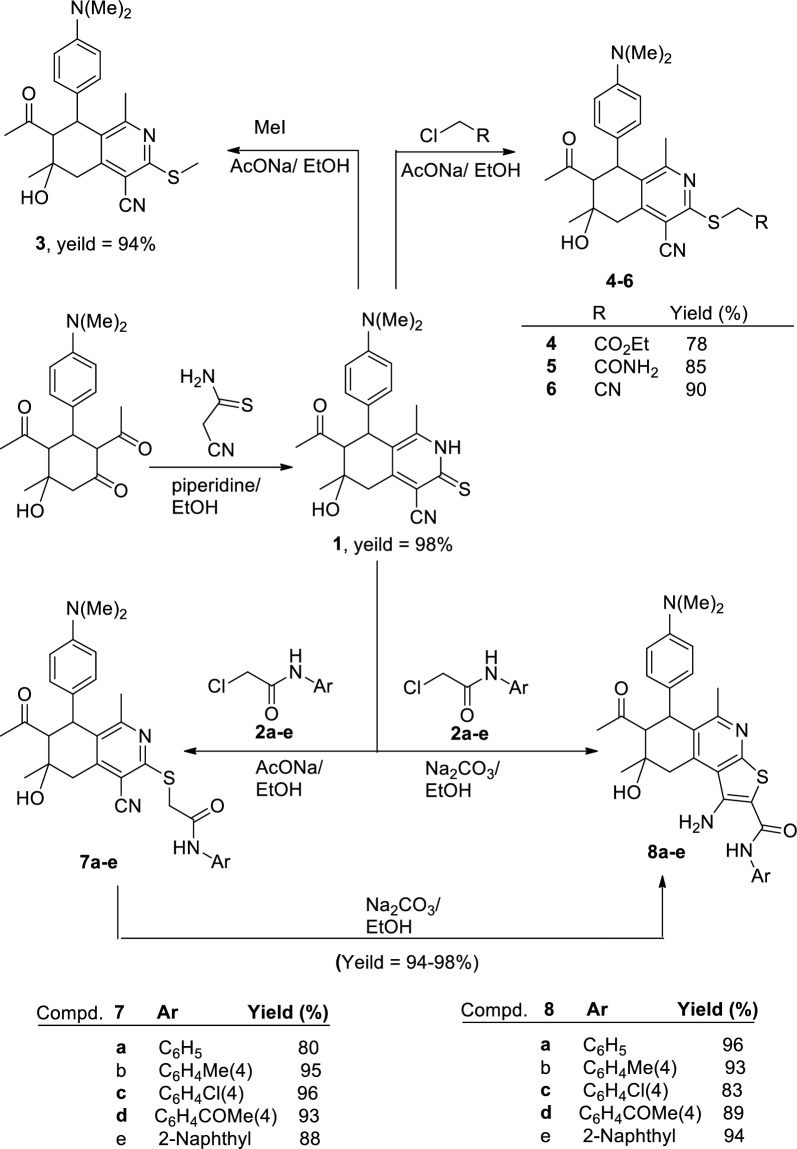
Synthesis of compounds **1,3–6,7a–e** and **8a–e**

Refluxing of 2,4-diacetyl-5-hydroxy-5-methyl-3-(4-(*N*,*N*-dimethylaminophenyl) cyclohexanone with 2-cyanothioacetamide in ethanol in the presence of piperidine as a basic catalyst resulted in regioselective cyclocondensation reaction affording, 7-acetyl-4-cyano-1,6-dimethyl-6-hydroxy-8-(4-(*N*,*N*-dimethylaminophenyl)-5,6,7,8-tetrahydroiso-quinoline-3(2*H*)-thione (**1)** in 98% yield (Scheme 1).

Compound **1** underwent *S*-alkylation reactions upon treatment with some halo reagents namely; methyl iodide, ethyl chloroacetate, 2-chloroacetamide, chloroacetonitrile or *N*-aryl-2-chloroacetamides **2a–e** in refluxing ethanol containing slightly excess molar amounts of sodium acetate trihydrate to give 3-ethylthio-5,6,7,8-tetrahydroisoquinoline **3**, ethyl (5,6,7,8-tetrahydroisoquinolin-3-ylthio)acetate **4**, (5,6,7,8-tetrahydroisoquinolin-3-ylthio)acetamide **5,** (5,6,7,8-tetrahydroisoquinolin-3-ylthio)acetonitrile **6** and 2-[(7-acetyl-4-cyano-1,6-dimethyl-6-hydroxy-8-(4-(*N*,*N*-dimethylaminophenyl)-5,6,7,8-tetrahydroisoquinolin-3-yl)thio]-*N*-arylacetamides **7a–e**, respectively (Scheme 1).

On heating of compounds **7a–e** with catalytic amounts of anhydrous sodium carbonate in abs. ethanol, they underwent intramolecular Thorpe-Ziegler cyclization affording 7-acetyl-1-amino-*N*-aryl-5,8-dimethyl-8-hydroxy-6-(4-*N*,*N*-dimethylamino-phenyl)-6,7,8,9-tetrahydrothieno[2,3-*c*]isoquinoline-2-carboxamides **8a–e** in nearly quantitative yield (Scheme 1). Compounds **8a–e** were also synthesized via reaction of **1** with the respective *N*-aryl-2-chloroacetamides **2a–e** by heating in abs. ethanol in the presence of slightly excess molar amounts of anhydrous sodium carbonate (Scheme 1).

### Characterization

The structures of all newly synthesized compounds were confirmed by FT-IR, ^1^H NMR and ^13^C NMR as well as elemental analyses (cf. Experimental part section and Additional file 1: Figs. S1–S45).

### Anticancer activities

#### In vitro cytotoxicity

**Fig. 1:**
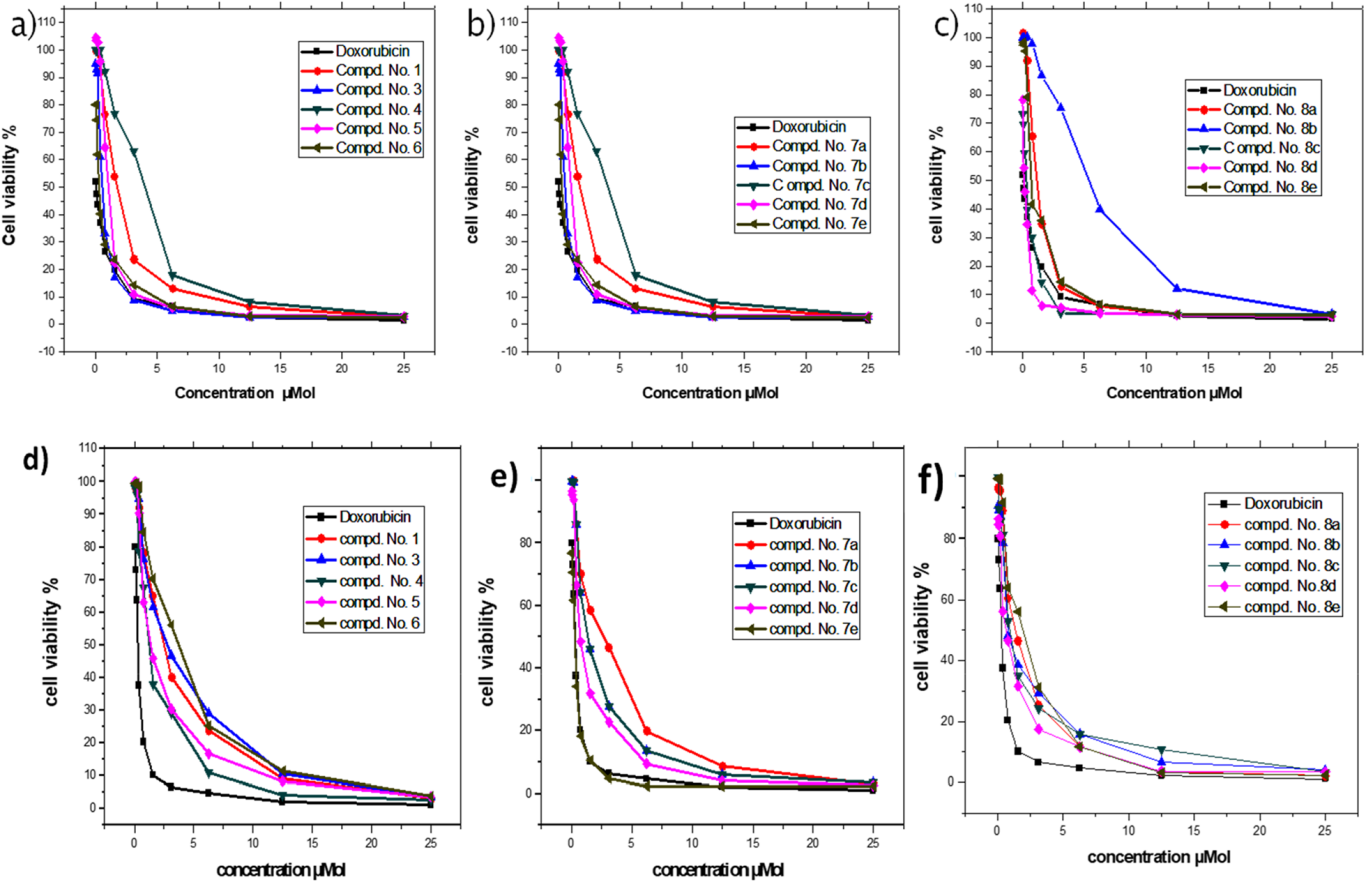
Anticancer activity of synthesized compounds compared with Doxorubicin as a standard at different concentrations from 0.048 to 25 μM. **a-** Compounds **1** and **3–6**. **b-** Compounds **7a–7e**. **c**- Compounds **8a–8e** against MCF7 cell line respectively. **d** -Compounds **1** and **3–6**. **e** -Compounds **7a–7e**.f -Compounds **8a–8e** against the A549 cell line respectively

Our newly synthesized compounds **1, 3–6, 7a–e,** and **8a–e** were studied for their in vitro cytotoxic activities against two selective cell lines MCF7 and A549 (which our compounds show high activities towards them by using a way to drug predication program) by using the MTT assay method [36, 37]. In this work, doxorubicin was used as a positive control drug for comparison with the synthesized compounds under the same experimental conditions. Ten concentrations of each compound and doxorubicin ranging from 0.04875 to 25 μM were tested to reach the concentration which could cause death for 50% of the cancer cells (IC50). The cell viability and toxicity percentage are given in supplementary data (Additional file 1: Tables S1–S6), and summarized in Table 1 and Fig. 1. These results indicated that all synthesized compounds possess high cytotoxic activity against the two cell lines under investigation compared with that of doxorubicin, with IC_50_ values ranging from 0.117 to 3.800 μM (Table 1).

**Table 1:** Cytotoxicity (IC_50_) of compounds **1**, **3–6, 7a–7e**, **8a**–**e** and doxorubicin as a standard against both **MCF7**, **A549** cell lines

Compound no.	MCF7 cell line	**A459** cell line
	IC_50_ ± SD (µM)	IC_50_ ± SD (µM)
**1**	1.857 ± 0.008	2.219 ± 0.002
**3**	0.562 ± 0.007	2.469 ± 0.006
**4**	3.074 ± 0.008	0.918 ± 0.002
**5**	0.924 ± 0.007	1.247 ± 0.002
**6**	0.329 ± 0.005	3.736 ± 0.002
**7a**	2.218 ± 0.004	1.586 ± 0.001
**7b**	0.474 ± 0.006	0.987 ± 0.002
**7c**	1.491 ± 0.004	0.496 ± 0.003
**7d**	0.495 ± 0.002	0.446 ± 0.004
**7e**	0.211 ± 0.002	0.155 ± 0.003
**8a**	0.872 ± 0.003	1.045 ± 0.006
**8b**	3.800 ± 0.008	0.527 ± 0.002
**8c**	0.215 ± 0.005	0.332 ± 0.002
**8d**	0.117 ± 0.004	0.515 ± 0.002
**8e**	0.461 ± 0.002	1.329 ± 0.004
Doxorubicin	0.053 ± 0.002	0.218 ± 0.005

In more details on structure–activity relationship, we noticed that: (i) the cytotoxic activity of compounds **1** and **3–6** against **MCF7** cells obeys the order **6** > **3** > **5** > **1** > **4,** whereas that of the same compounds obeys approximately opposite order against **A549** cells as** 4** > **5** > **1** > **3** > **6;** (ii) 4-substituted phenylcarbamoylmethylthio derivatives **7b–d** exhibited stronger cytotoxic activity than the parent unsubstituted one **7a** against both **MCF7** and **A549** cell lines; (iii) among the arylcarbamoylmethylthioisoquinolines **7a–e** and arylcarbamoyl thienoisoquinolines **8a–e**, naphalen-1-yl derivative **7e** exhibited the highest cytotoxic activity against A549 cell line and 4-chlorophenyl derivative **8d** showed the highest activity against MCF7 cell line, respectively. Moreover the toxicity of these two compounds against normal human fetal lung fibroblast *WI*-*38* cell line were investigated in this study which show that **7e** and **8d **compounds not toxic and safe for normal lung cell line with IC_50_ 19.7 µM, 23.3 µM respectively in comparison with Doxorubicin  IC_50_ 11.43 µM (Table 2) the test details presented in Additional file 1: Table S6a.

**Table 2:** Cytotoxicity (IC_50_) of compounds **7e** and **8d** and Doxorubicin against normal cell line **WI-38** cell line

Code	Toxicity on WI38 IC50 µM ± SD	Selectivity index (SI)
**7e**	19.734 ± 0.79	127.29
**8d**	23.301 ± 0.93	45.24
Doxorubicin	11.433 ± 0.37	52.4

By calculating the selectivity index of these compounds **7e, 8d** and Doxorubicin ((SI) = IC_50_ of compound in noncancerous cell line (WI-38)\IC_50_ of compound in cancer cell (A549)). They show very high selectivity index SI = 127, 45, 52 respectively. therefore these compounds belong of a selected potential anticancer drugs.Cyclization of arylcarbomyl-methylthioisoquinolines **7a** and **7c** into the corresponding arylcarbomylthienoiso-quinolines **8a** and **8c** resulted in increasing the anticancer activity towards both **MCF7** and **A549** cell lines; (v) cyclization of tolylcarbomylmethylthioisoquinolines **7b** into the corresponding tolylcarbomylthieno[2,3-c]isoquinolines **8b** decreases the anticancer activity towards **MCF7** cell line and (vi) cyclization of carbomylmethylthioisoquinolines **7e** into the corresponding carbomylthienoisoquinoline **8e** decreases the anticancer activity towards both **MCF7** and **A549** cell lines (Fig. 1, and Table 1).

#### Cell cycle analysis in MCF7 and A549 Cells

**Fig. 2:**
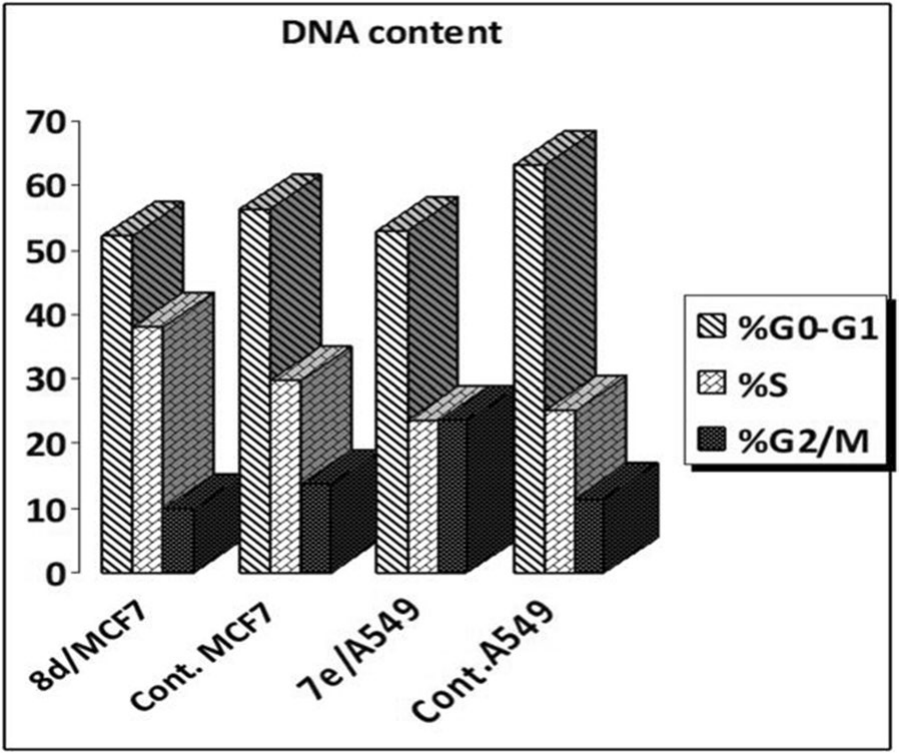
Cell cycle analysis of **A549** and **MCF7** cells treated with compounds **7e** and **8d**

The high cytotoxic activity of compound **7e** against **A549** (IC_50_ 0.155 µM) and compound **8d** against the **MCF7** cell line (IC_50_ 0.170 µM) prompted us to further investigate the growth inhibitory mechanism of the target conjugates to study the mechanism of the cell cycle by using flow cytometric analysis [46–48]. Both regulation of cell cycle progression and apoptosis induction have been considered significant strategies to control the proliferation of different cancer cells, accordingly, we primarily examined the growth inhibition mechanism of compounds **7e** and **8d** in relation to cell cycle progression and regulation in **A549** and **MCF7** cancer cells, respectively (Fig. 2, and Table 3).

**Table 3:** Cell cycle analysis of A549 and MCF7 cells treated with compounds **7e** and **8d**

Sample code	DNA content		
	%G0-G1	%S	%G2/M
**8d/**MCF7	52.03	37.92	10.05
Cont. MCF7	56.42	29.81	13.77
**7e**/A549	52.83	23.56	23.61
Cont. A549	63.29	25.11	11.6

The impact on cell cycle distribution was assessed by a DNA flow cytometry analysis, through incubation of **A549** cells with compound **7e** at its IC_50_ concentration (IC_50_ 0.155 µM) and incubation of **MCF7** cells with compound **8d** at its IC_50_ concentration (IC_50_ 0.170 µM) for 48 h (Fig. 2). From the obtained results, it was found that: (i) **A549** cells exposed to compound **7e** significantly arrested at the G2/M phase of the cell cycle with an escalation in G2/M phase fraction from 11.60 (in control cells) to 23.61% (in **7e**-treated **A549** cells) and (ii) **MCF7** cells treated with compound **8d** had a significant decrease in G0-G1 and G2/M phases than control cells in contrast (iii) S phase was significantly increased in treated cells as an indication of cell cycle arrest; i.e. increased from 29.81 (control) to 37.92 (**8d**-treated cells). The antiproliferative mechanism of our compounds was explored from the aforementioned obtained result; compounds of type **7e** compound arrested the cell cycle at G2/M phase of the cell cycle whereas compounds of type **8d** compound arrested the cell cycle at S phase (Fig. 2).

#### Apoptosis assay in A549 and MCF7 cell lines

**Fig. 3:**
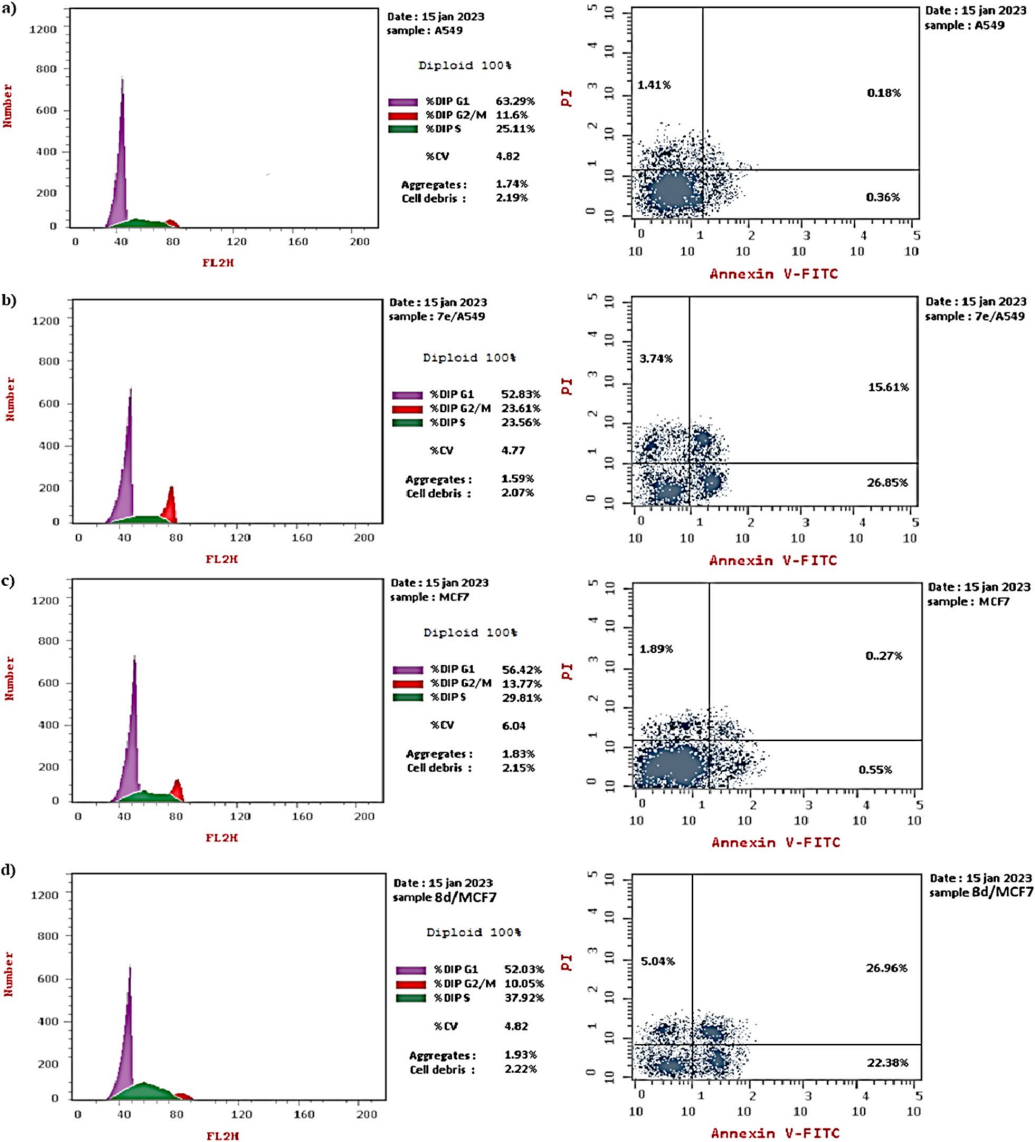
Apoptosis results of compounds **7e** and **8d** on A549 and MCF7 cell lines respectively. **a**. Control A549 ** b**. Compd. **7e** \A549 and **c**. MCF7 control. **d**. Compd. **8d\MCF7**

To further investigate whether the anti-proliferative activity for compound **7e** or **8d** is harmonious with the apoptosis induction [47–50] within **A549** or **MCF7** cells pointed out by the increased cell population in G2/M phase in **7e**-treated **A549** cells and S phase in **8d**-treated **MCF7** cells, respectively, and AnnexinV-FITC/PI dual staining analysis was used for the apoptosis assay (Fig. 3).

**Fig. 4:**
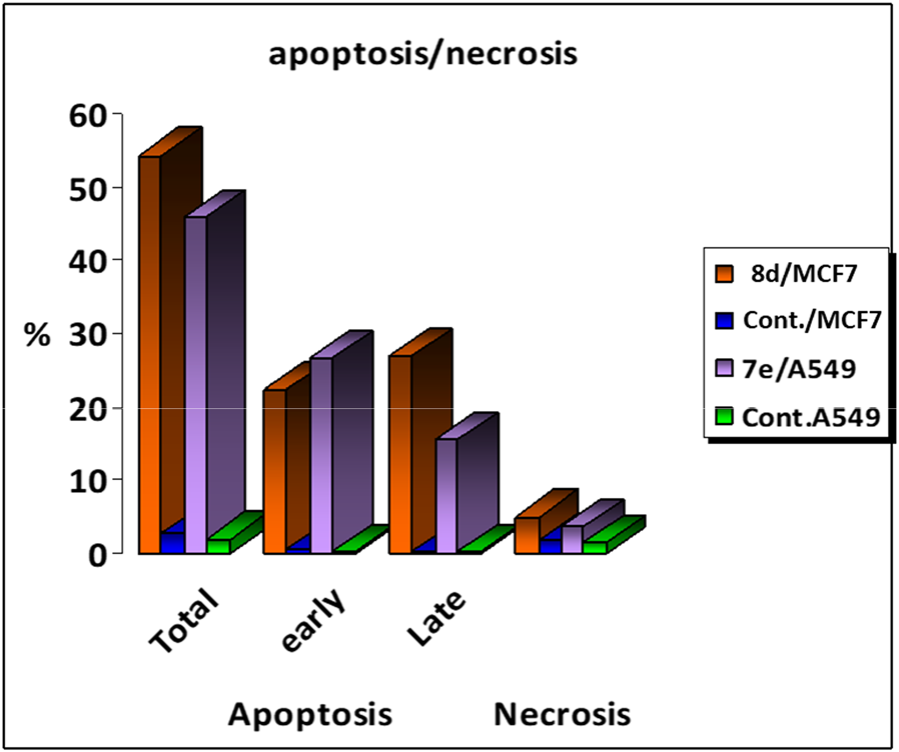
Apoptosis/necrosis assessment of **A549** and **MCF7** cells after treatment with compounds **7e** against **A549** and **8d** against **MCF7**. Different cell populations were plotted as a percentage of total events. Data are presented as mean ± SD; n = 3

The results of the Annexin V-FITC/PI assay suggested that: (i) treatment of **A549** cells with compound **7e** led to early and late cellular apoptosis, which proved through the significant increase the percentage of the apoptotic cells in both the early apoptotic phase (from 0.36 to 26.85%) and the late apoptotic phase (from 0.18 to 15.61%) that indicates a high increase in total apoptosis when compared to the untreated control (Fig. 3a, b), (ii) compound **8d** caused a considerable increase in early and late apoptosis of **MCF7** cells than control cells; i.e. the early and late apoptotic population increased from 0.55 to 22.38% and from 0.27 to 26.96%, respectively (Fig. 3c, d), and (iii) treating **A549** cells with compound **7e** increases the population of necrotic cell from 1.41 (control) to 3.73% keeping the necrosis minimally contributing. Also, the population of necrotic cells increases from 1.89 (control) to 5.04% upon the subjection of the **MCF7** cells to compound **8d** (Fig. 4). From the above results, an overall 79-fold increase in **A549** cellular apoptosis after treatment with compounds **7e** and 69-fold increase in MCF7 cellular apoptosis after treatment with 8d compound In comparison to the control. We observed that our targeted substances, **7e** and **8d**, have the potential to function as a biological mechanism for inhibiting cell growth, thus leading to cytotoxic effects against the **MCF7** and **A549** cell line (Fig. 4).

#### Molecular docking

**Fig. 5:**
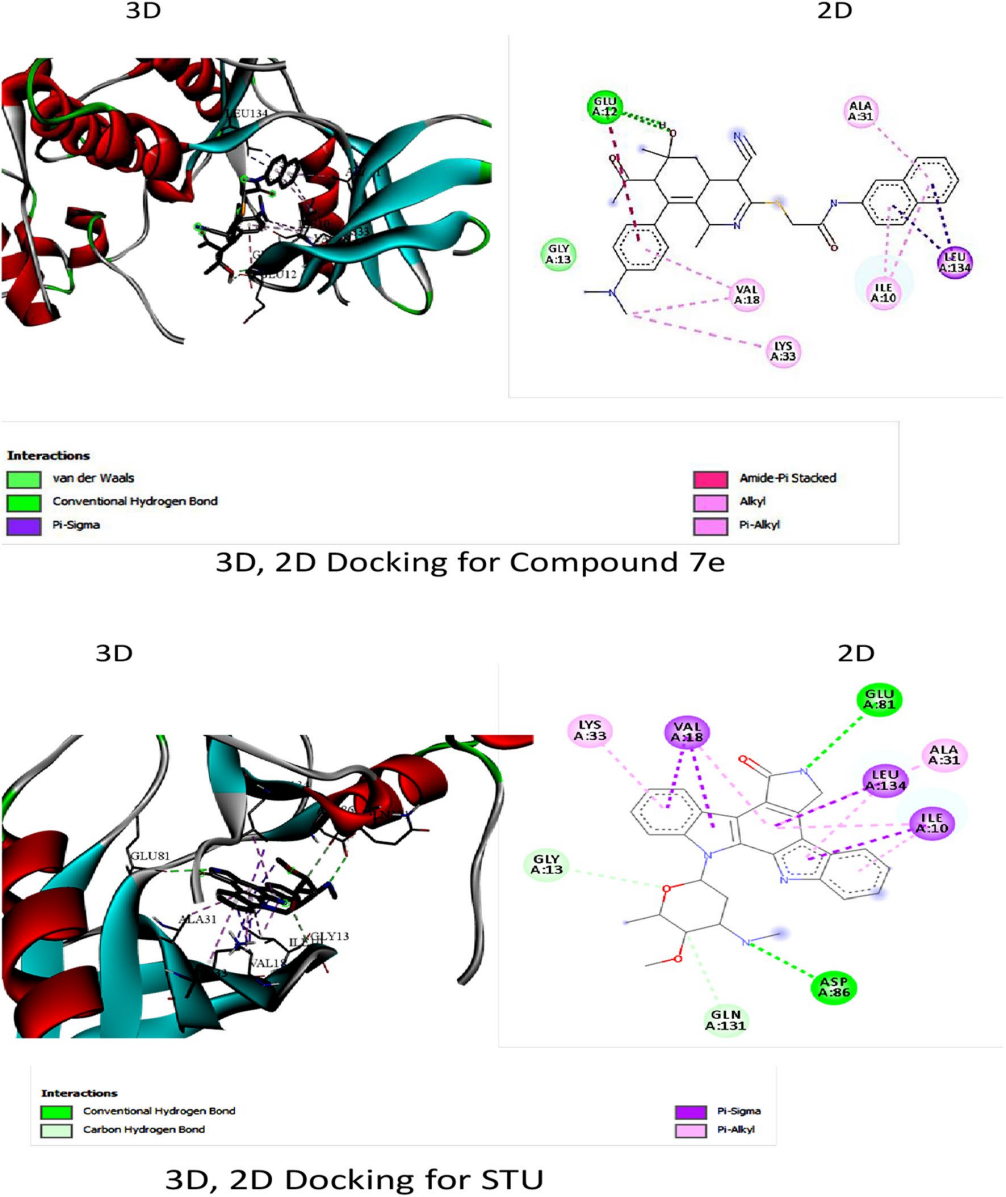
3D and 2D docking interaction of compound **7e** with CDK2 in compered to the slandered STU299

The docking studies revealed that compound **7e** had stronger binding affinity (− 10.3 kcal/mol) to CDK2 compared to the standard STU299 (− 11.5 kcal/mol). The interactions analysis showed that **7e** formed hydrogen bonds, amid pi-sulfate, alkyl, pi-alkyl, and pi-sigma interactions with key amino acid residues in the CDK2 binding site like GLU 12, VAL 18, LYS 33, and LEU 134 (Table 4, Fig. 5). In contrast, STU299 showed hydrogen bonds, C–H bonds, alkyl, pi-alkyl, and pi-sigma interactions with residues like GLY 13, GLN 131, LEU 134, VAL 18, ILE 10. The additional pi-sulfate and amid interactions of **7e** with GLU 12 likely contribute to its better binding over STU299.

**Table 4:** ∆G and binding affinity (Kcal/mol) for CDK2 docking interaction with compound **7e** in comparison its standard stu299

Compound	∆G and binding affinity (Kcal/mol)
**7e**	− 10.3
STU299	− 11.5

**Fig. 6:**
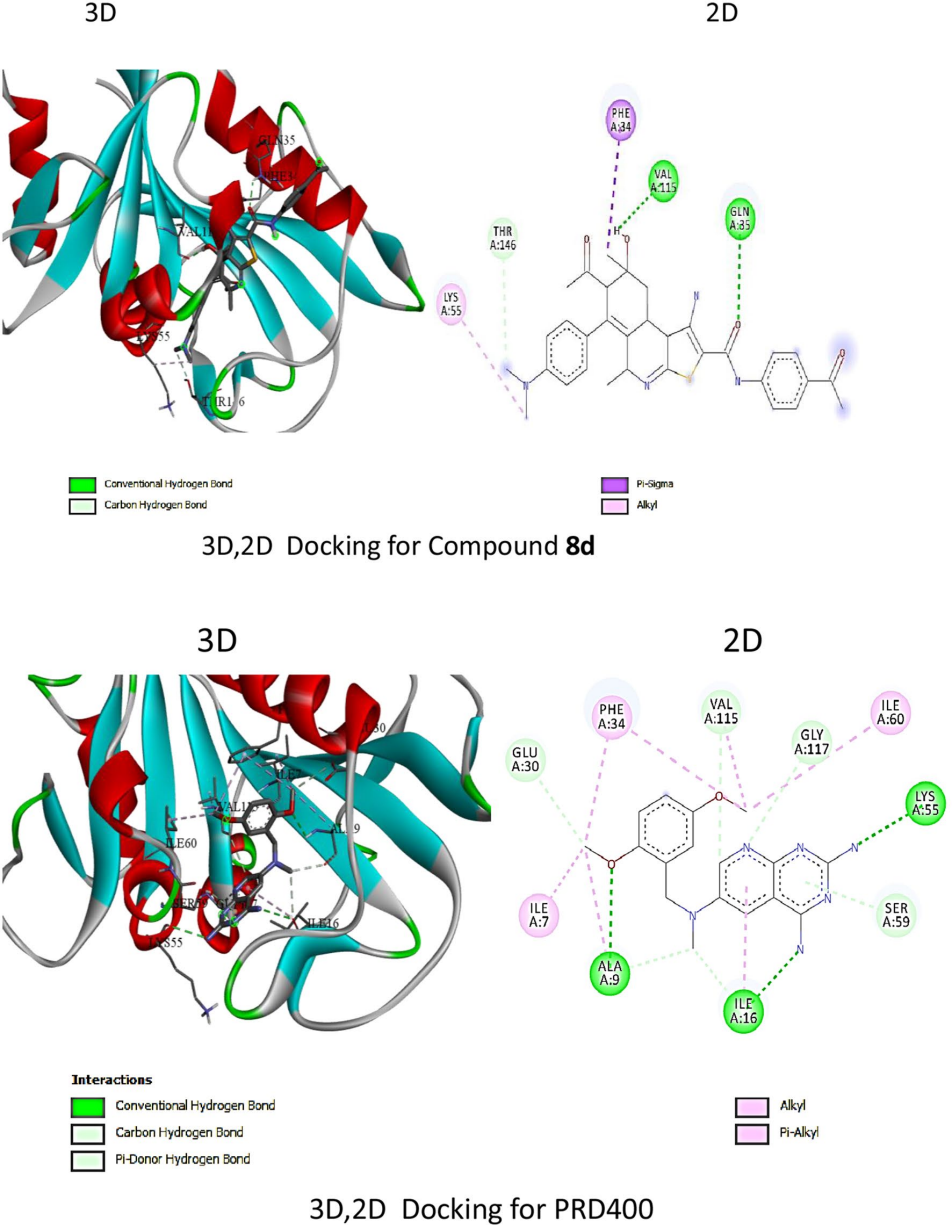
3D and 2D docking interaction of compound **8d** with DHFR in compered to the slandered PRD400

For DHFR, compound **8d** had a stronger binding affinity (− 9.5 kcal/mol) than the standard PRD400 (− 8.5 kcal/mol). The interactions analysis revealed **8d** forms hydrogen bonds, C–H bonds, alkyl, and pi-sigma interactions with key residues like VAL 115, GLN 35, PHE 34 in the DHFR binding site (Table 5, Fig. 6). Meanwhile, PRD400 showed hydrogen bonds, C–H bonds, alkyl, and pi-alkyl interactions with residues such as LYS 55, ALA 9, ILE 16, SER 59, GLY 117, ILE 7, PHE 34. The extra pi-sigma interaction of **8d** with PHE 34 may enhance its binding over PRD400.

**Table 5:** ∆G and binding affinity (Kcal/mol) for DHFR docking with **8d** with its standard PRD400

Compound	∆G and binding affinity (Kcal/mol)
**8d**	− 9.5
PRD400	− 8.5

Overall, the docking results indicate compounds **7e** and **8d** bind more strongly to CDK2 and DHFR respectively compared to the standard inhibitors. The additional interactions formed by **7e** and **8d** with key active site residues likely contribute to their enhanced binding affinity.

#### Enzyme inhibitory activities

The promising anti-proliferative impact of compounds **7e** and **8d**, in addition to their cell cycle disruption and pro-apoptotic effects, proved a further exploration for their possible inhibitory activities against many enzymes such as RET (encodes a receptor tyrosine kinase) and **CDK2** (Cyclin-dependent kinase 2) treated with compound **7e**, and **DHFR (**Dihydrofolate reeducates), **Eef2** Kinase (Eukaryotic elongation factor 2kinase)and **IKB** kinase (inhibitory kappa B kinase) treated with compound **8d**.

##### Inhibitory activity of compound 7e towards CDK2

**Fig. 7:**
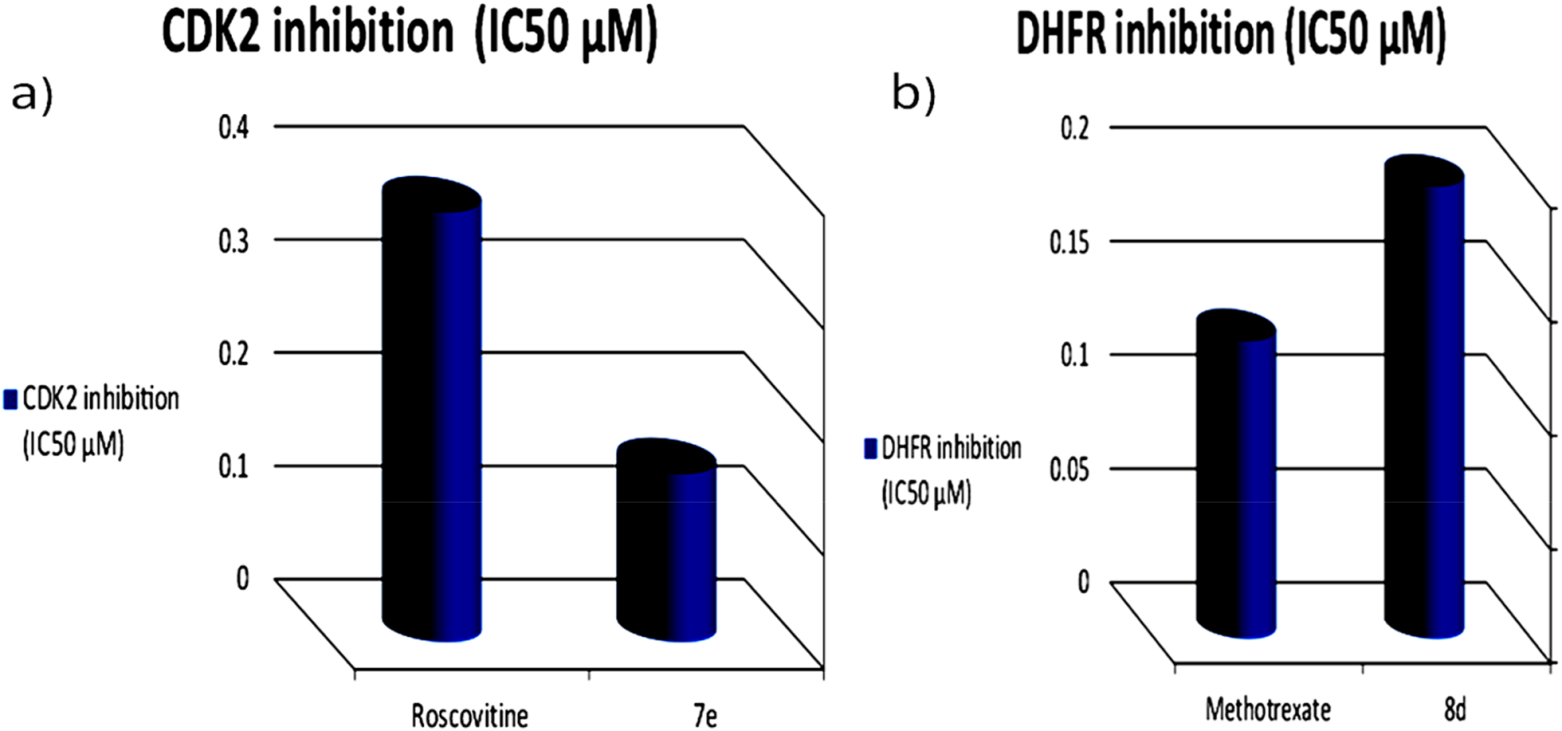
**a-  CDK2 inhibitory activity of compound 7e. b- DHFR** inhibitory activity of compound **8d**.

Compound **7e** showed significant **CDK2** cyclin A inhibitory activity in comparison with the reference; Roscovitine Table 6. Due to the nature of isoquinoline moiety [51, 52]. From the docking study the inhibition mechanism of compound **7e** with interaction with CDK2 with hydrogen bonding and other bonds; they may deactivate the binding site in CDK2 and either its partners or substrates resulting in specific inhibition of CDK2. The obtained results in Table 6 and Fig. 7a and for more enzyme inhibition test details presented in supplementary data (Additional file 1: Table S7) showed that the tested compound **7e** exhibited significant inhibitory action against CDK2 with IC_50_ value 0.149 ± 0.007 in comparison with the control; Roscovitine which showed IC_50_ of 0.380 ± 0.008 µM (reference of CDK2 inhibitor).

**Table 6:** **CDK2** inhibitory activity of compound **7e**

Compd. no.	M.W. (g/mol)	CDK2 inhibition (IC_50_ ± SD; µM)
**7e**	578	0.149 ± 0.007
Roscovitine	354.5	0.380 ± 0.008

##### DHFR inhibitory activity of compound 8d

Our results obtained indicated that compound **8d** which contains tetrahydrothieno[2,3-c]isoquinoline [14, 53, 54] moiety showed high inhibitory activity towards DHFR enzyme in comparison with the reference; methotrexate show Table 7, Fig. 7b and for more enzyme inhibition test details was presented in supplementary data (Additional file 1: Table S8). Thus, compound **8d** exhibited good inhibitory activity towards DHFR with IC_50_ value 0.199 ± 0.016 in comparison with that Methotrexate (IC_50_ of 0.131 ± 0.007).

**Table 7:** **DHFR** inhibitory activity of the compound **8d**

Compd. no.	M.W. (g/mol)	DHFR inhibition (IC_50_ ± SD; µM)
**8d**	670	0.199 ± 0.016
Methotrexate	454.44	0.131 ± 0.007

##### Other enzyme inhibitory activity

Compounds**7e** and **8d** exhibited moderate inhibitory activity towards other enzymes under investigation in comparison with their control for more enzyme inhibition test details show Additional file 1 (Table 8 and Additional file 1: Tables S9–S11).

**Table 8:** Enzyme inhibitory activity of compounds **7e** and **8d**

**RET** tyrosine kinase (**IC**_**50**_ ± SD; µM)		**Eef2** kinase (**IC**_**50**_ ± SD; µM)		**IKB** kinase B (**IC**_**50**_ ± SD; µM)	
Compound** 7e**	Control (staurosporine)	Compound** 8d**	Control (NH125)	Compound **8d**	Control (TPCA-1)
0.106 ± 0.005	0.069 ± 0.003	0.689 ± 0.036	0.357 ± 0.0190	0.240 ± 0.013	0.072 ± 0.004

### In vitro antioxidant behavior

**Fig. 8:**
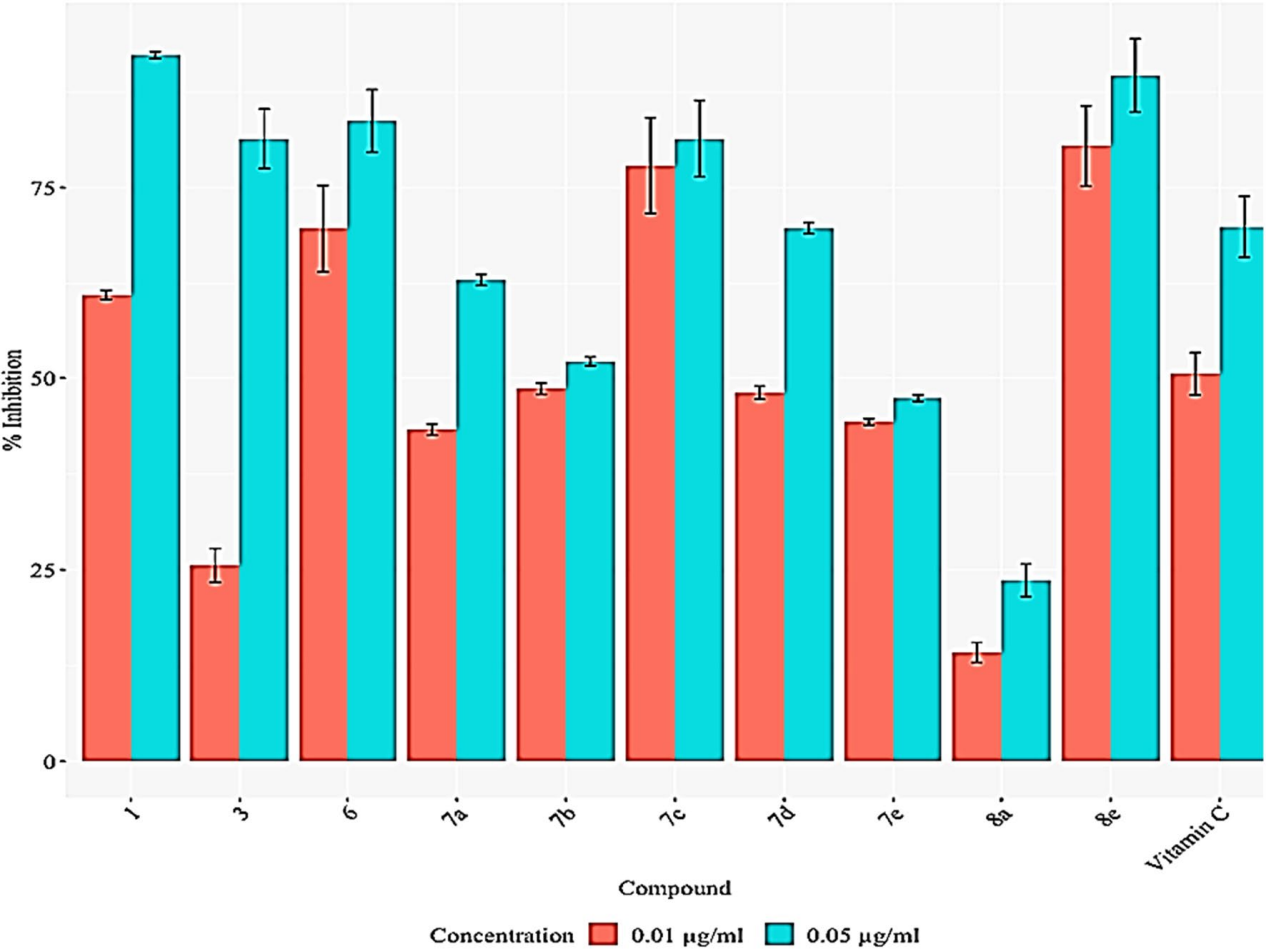
Antioxidant activity of compounds **1**, **3**, **6**, **7a**, **7b**, **7c**, **7d**, **7e**, **8a** and **8e**

Ten newly synthesized compounds were studied as in vitro antioxidants by measuring of their DPPH scavenging activity which is represented as a percentage % [32]^.^ Results are represented by mean ± SD of three replicates. Table 9 showed the percentage of DPPH scavenging activity of the tested compounds in a dose-dependent relationship compared with Vitamin C (ascorbic acid) as a standard. The higher dose concentration of 0.05 μg/mL represents higher antioxidant activity. Compounds **1**, **3**, **6**, **7c** and **8e** have higher result than Vitamin C itself. Compound **8e** show the highest significant result which suggests that this compound can be used as excellent antioxidant drugs. The high antioxidant activity is referred to the presence of C=O, NH_2_, and OH groups like ascorbic acid [55, 56] which can be easily oxidized and reduced and can be used as antioxidant drugs. (Fig. 8 and Table 9).

**Table 9:** DPPH Scavenging activity of 5,6,7,8-tetrahydrothieno[2,3-c]isoquinolines 1, 3, 6 and **7a**–**e**, and 6,7,8,9-tetrahydrothieno[2,3-c]isoquinolines **8a**, **b**

Compound no.	0.01 µg/mL inhibition (%)	0.05 µg/mL inhibition (%)
**1**	61.01 ± 0.58	92.3 ± 0.44
**3**	25.58 ± 2.20	81.39 ± 3.87
**6**	69.67 ± 5.65	83.72 ± 4.08
**7a**	43.26 ± 0.73	62.96 ± 0.73
**7b**	48.59 ± 0.73	52.19 ± 0.58
**7c**	77.90 ± 6.22	81.39 ± 4.99
**7d**	48.08 ± 0.87	69.73 ± 0.73
**7e**	44.28 ± 0.44	47.36 ± 0.44
**8a**	14.22 ± 1.32	23.66 ± 2.12
**8e**	80.45 ± 5.22	89.67 ± 4.76
Vitamin C	50.54 ± 2.76	69.90 ± 3.98

*These data are represented by Mean ± SD. DPPH scavenging activity represented as %. Statistical analysis is carried out using two-way ANOVA coupled with a CO-state computer. The ascorbic acid standard was used as a positive control. DPPH scavenging activity was calculated as follows: % Inhibition = 100 − [Absorbance of the test compound/Absorbance of the control] × 100

The important of the information in the asterisk : to inform the software (ANOVA) used in this study and the equation used for calculation the results

## Conclusion

In this paper, We successfully synthesized and characterized of novel two series of substituted methylthiotetrahydroisoquniolines and related tetrahydrothieno[2,3-c]isoquinolines. All synthesized compounds were evaluated for their anticancer activity towards A549 and MCF7 cell lines, and showed promising results. Moreover, the cell cycle arrest and apoptosis induction of the two representative compounds was studied. Compound **7e** caused cell cycle arrest of A549 cell line at G2/M phase and compound **8d** arrest the cell cycle of MCF7 cell line at S phase. Compounds **7e** and **8d** compounds caused high increase in the early and late apoptosis and necrosis. Furthermore, compound **7e** showed significant inhibition of CDK2 enzyme while compound **8d** exhibited significant activity as a DHFR inhibitors. In the future we intend to synthesis new series of tetrahydrothieno[2,3-c]isoquinolines to studied their anticancer activity not only in vitro but also in vivo and examined the anticancer activity of these compounds in patient samples as potent anticancer drugs.

### Supplementary Information

**Additional file 1: Fig. S1**. FT-IR spectrum of Compound **1**. **Fig. S2**. ^1^H NMR spectrum of Compound **1**. **Fig. S3**. ^13^C NMR spectrum of compound **1**. **Fig. S4**. FT-IR spectrum of compound **3**. **Fig. S5**. ^1^H NMR spectrum of compound **3**. **Fig. S6**. ^13^C NMR spectrum of compound **3**. **Fig. S7**. FT-IR spectrum of compound **4**. **Fig. S8**. ^1^H NMR spectrum of compound **4**. **Fig. S9**. ^13^C NMR spectrum of compound **4**. **Fig. S10**. FT-IR spectrum of compound **5**. **Fig. S11**. ^1^H NMR spectrum of compound **5**. **Fig. S12**. ^13^C NMR spectrum of compound **5**. **Fig. S13**. FT-IR spectrum of compound **6**. **Fig. S14**. ^1^H NMR spectrum of compound **6**. **Fig. S15**. ^13^C NMR spectrum of compound **6**. **Fig. S16**. FT-IR spectrum of compound **7a**. **Fig. S17**. ^1^H NMR spectrum of compound **7a**. **Fig. S18**. ^13^C NMR spectrum of compound **7a**. **Fig. S19**. FT-IR spectrum of compound **7b**. Fig. **S20**. ^1^H NMR spectrum of compound **7b**. Fig. **S21**. ^13^C NMR spectrum of compound **7b**. Fig. **S22**. FT-IR spectrum of compound **7c**. **Fig. S23**. ^1^H NMR spectrum of compound **7c**. **Fig. S24**. ^13^C NMR spectrum of compound **7c**. **Fig. S25**. FT-IR spectrum of compound **7d**. **Fig. S26**. ^1^H NMR spectrum of compound **7d**. **Fig. S27**. ^13^C NMR spectrum of compound **7d**. **Fig. S28**. FT-IR spectrum of compound **7e**. **Fig. S29**. ^1^H NMR spectrum of compound **7e**. **Fig. S30**. ^13^C NMR spectrum of compound **7e**. **Fig. S31**. FT-IR spectrum of compound **8a**. **Fig. S32**. ^1^H NMR spectrum of compound **8a**. **Fig. S33**. ^13^C NMR spectrum of compound **8a**. **Fig. S34**. FT-IR spectrum of compound **8b**. **Fig. S35**. ^1^H NMR spectrum of compound **8b**. **Fig. S36**. ^13^C NMR spectrum of compound **8b**. **Fig. S37**. FT-IR spectrum of compound **8c**. **Fig. S38**. ^1^H NMR spectrum of compound **8c**. **Fig. S39**. ^13^C NMR spectrum of compound **8c**. **Fig. S40**. FT-IR spectrum compound **8d**. **Fig. S41**. ^1^H NMR spectrum compound **8d**. **Fig. S42**. ^13^C NMR spectrum of compound **8d**. **Fig. S43**. FT-IR spectrum compound **8e**. **Fig. S44**. ^1^H NMR spectrum compound **8e**. **Fig. S45**. ^13^C NMR spectrum of compound **8e**. **Table S1**. Raw date of toxicity and viability of compounds **1**,**3**–**6** against MCF7. **Table S2**. Raw date of toxicity and viability of compounds **7a**–**e** against MCF7. **Table S3**. Raw date of toxicity and viability of compounds **8a**–**e** against MCF7. **Table S4**. Raw date of toxicity and viability of compounds **1**,**3**–**6** against A549. **Table S5**. Raw date of toxicity and viability of compounds **7a**–**e** against A549. **Table S6**. Raw date of toxicity and viability of compounds **8a**–**e** against A549. **Table S7**. **CDK2** inhibitor detailed results. **Table S8**. **DHFR** inhibitor detailed results. **Table S9**. **Eef2** inhibitor detailed results. **Table S10**. **IKB** inhibitor detailed results. **Table S11**. **RET** inhibitor detailed results.

## Data Availability

All data generated or analyzed during this study are in this published article and supplementary information.
