# Supplementary material for: Novel tetrahydroisoquinolines as DHFR and CDK2 inhibitors: synthesis, characterization, anticancer activity and antioxidant properties
Source: BMC Chem. 2024 Feb 16;18(1):34. doi: 10.1186/s13065-024-01139-w (PMC10873978; doi:10.1186/s13065-024-01139-w)

**Supporting information**

**Novel tetrahydroisoquinolines as DHFR and CDK2 inhibitors: Synthesis, characterization, anticancer activity and antioxidant properties**

**Eman M. Sayed,^a^ Etify A. Bakhite,^b^* Reda Hassanien,^a^ Nasser Farhan,^a^ Hanan F. Aly,^c^ Salma G. Morsy,^d^ Nivin A. Hassan^e^**

^a^Chemistry Department, Faculty of Science, New Valley University, El-Kharja 72511, Egypt. ^b^Chemistry Department, Faculty of Science, Assiut University, Assiut 71516, Egypt. ^c^Department of Therapeutic Chemistry, National Research Centre, El-Behooth St., 12622 Dokki, Cairo, Egypt. ^d^Department of Cancer Biology (Cancer Immunology and Virology), South Egypt Cancer Institute, Assiut University, Assiut, Egypt. ^e^Pharmacology and Experimental Oncology Unit, Cancer Biology Department, South Egypt Cancer Institute, Assiut University, Assiut, Egypt.

*Corresponding author

Email: emysayed880@yahoo.com

**Table of Contents**

| **Figure S1**: FT-IR spectrum of compound 1. |  |
| --- | --- |
| **Figure S**2: ^1^H NMR spectrum of compound 1. |  |
| **Figure S3**: ^13^ C NMR spectrum of compound 1. |  |
| **Figure S4**: FT-IR spectrum of compound 3. |  |
| **Figure S5**: ^1^H NMR spectrum of compound 3. |  |
| **Figure S6**: ^13^ C NMR spectrum of compound 3. |  |
| \| **Figure S7**: FT-IR spectrum of compound 4. \|  \| \| --- \| --- \| \| **Figure S8**: ^1^H NMR spectrum of compound 4. \|  \| \| **Figure S9**: ^13^ C NMR spectrum of compound 4. \|  \| \| **Figure S10**: FT-IR spectrum of compound 5  **Figure S11**: ^1^H NMR spectrum of compound 5. \|  \| |  |
|  |  |
| **Figure S12**: ^13^ C NMR spectrum of compound 5. |  |
| **Figure S13**: FT-IR spectrum of compound 6. |  |
| **Figure S14**: ^1^H NMR spectrum of compound 6. |  |
| **Figure S15**: ^13^ C NMR spectrum of compound 6. |  |
| **Figure S16**: FT-IR spectrum of compound 7a. |  |
| **Figure S17**: ^1^H NMR spectrum of compound 7a . |  |
| **Figure S18**: ^13^ C NMR spectrum of compound 7a. |  |
| **Figure S19**: FT-IR spectrum of compound 7b. |  |
| **Figure S20**: ^1^H NMR spectrum of compound 7b . |  |
| **Figure S21**: ^13^ C NMR spectrum of compound 7b. |  |
| **Figure S22**: FT-IR spectrum of compound 7c. |  |
| **Figure S23:**^1^H NMR spectrum of compound 7c |  |
| **Figure S24**: ^13^ C NMR spectrum of compound 7c. |  |
| **Figure S25**: FT-IR spectrum of compound 7d. |  |
| **Figure S26**: ^1^H NMR spectrum of compound 7d. |  |
| **Figure S27**: ^13^ C NMR spectrum of compound 7d. |  |
| **Figure S28**: FT-IR spectrum of compound 7e. |  |
| **Figure S29**: ^1^H NMR spectrum of compound 7e. |  |
| **Figure S30**: ^13^ C NMR spectrum of compound 7e. |  |
| **Figure S31**: FT-IR spectrum of compound 8a. |  |
| **Figure S32:** ^1^H NMR spectrum of compound 8a^.^ |  |
| **Figure S33**: ^13^ C NMR spectrum of compound 8a. |  |
| **Figure S34**: FT-IR spectrum of compound 8b. |  |
| **Figure S35**: ^1^H NMR spectrum of compound 8b. |  |
| **Figure S36**: ^13^ C NMR spectrum of compound 8b. |  |
| **Figure S37**: FT-IR spectrum of compound 8c. |  |
| **Figure S38:** ^1^H NMR spectrum of compound 8c. |  |
| **Figure S39**: ^13^ C NMR spectrum of compound 8c**.** |  |
| **Figure S40**: FT-IR spectrum compound 8d. |  |
| **Figure S41**: ^1^H NMR spectrum compound 8d. |  |
| **Figure S42**: ^13^ C NMR spectrum of compound 8d. |  |
| **Figure S43**: FT-IR spectrum compound 8e.   \| **Figure S44**: ^1^H NMR spectrum compound 8e . \|  \| \| --- \| --- \| \| **Figure S45**: ^13^ C NMR spectrum of compound 8e.  **Table S1**: Raw date of toxicity and viability of compds 1, 3-6 against MCF7. \| \| \| \|  \| |  |

**Table S2**: Raw date of toxicity and viability of compds 7a-e against MCF7.

**Table S3**: Raw date of toxicity and viability of compds 8a-e against MCF7.

**Table S4**: Raw date of toxicity and viability of compds 1, 3- 6 against A549

**Table S5**: Raw date of toxicity and viability of compds 7a-e against A549

**Table S6**: Raw date of toxicity and viability of compds 8a-e against A549.

**Table S7**: **CDK2** inhibitor detailed results.

**Table S8**: **DHFR** inhibitor detailed results.

**Table S9**: **Eef2** inhibitor detailed results.

**Table S10**: **IKB** inhibitor detailed results.

**Table S11**: RET inhibitor detailed results.

**Figure S1**: IR Spectrum of **7-Acetyl-4-cyano-1,6-dimethyl-6-hydroxy-8-(4-N,N-dimethylaminophenyl)-5,6,7,8-tetra-hydrosoquinoline-3(2*H*)-thione (1)**

**Figure S2:** ^1^H NMR Spectrum of **7-Acetyl-4-cyano-1,6-dimethyl-6-hydroxy-8-**

**.(4-N,N-dimethylaminophenyl)-5,6,7,8-tetra-hydrosoquinoline-3(2*H*)-thione (1)**

**Figure S3: ^13^**C NMR Spectrum of **7-Acetyl-4-cyano-1,6-dimethyl-6-hydroxy-8-(4-N,N-dimethylaminophenyl)-5,6,7,8-tetra-hydrosoquinoline-3(2*H*)-thione (1).**

**Figure S4**: IR Spectrum of **7-Acetyl-4-cyano-1,6-dimethyl-3-methylthio-6-hydroxy-8-**

**(4-N,N-dimethylamino-phenyl)-5,6,7,8-tetrahydroisoquinoline (3).**

**Figure S5:** ^1^H NMR Spectrum of **7-Acetyl-4-cyano-1,6-dimethyl-3-methylthio-6-hydroxy-8-(4-N,N-dimethylamino-phenyl)-5,6,7,8-tetrahydroisoquinoline (3).**

**Figure S6: ^13^**C NMR Spectrum of **7-Acetyl-4-cyano-1,6-dimethyl-3-methylthio-6-hydroxy-8-(4-N,N-dimethylamino-phenyl)-5,6,7,8-tetrahydroisoquinoline (3).**

**Figure S7**: IR Spectrum of **Ethyl 2-((7-Acetyl-4-cyano-1,6-dimethyl-6-hydroxy-8-(4-N,N-dimethylamino-phenyl)-5,6,7,8-tetrahydroisoquinolin-3-yl)thio)acetate** **(4).**

**Figure S8:** ^1^H NMR Spectrum of **Ethyl 2-((7-Acetyl-4-cyano-1,6-dimethyl-6-hydroxy-8-(4-N,N-dimethylamino-phenyl)-5,6,7,8-tetrahydroisoquinolin-3-yl)thio)acetate** **(4).**

**Figure S9: ^13^**C NMR Spectrum of **Ethyl 2-((7-Acetyl-4-cyano-1,6-dimethyl-6-hydroxy-8-(4-N,N-dimethylamino-phenyl)-5,6,7,8-tetrahydroisoquinolin-3-yl)thio)acetate** **(4).**

**Figure S10**: IR Spectrum of **2-[(7-Acetyl-4-cyano-1,6-dimethyl-6-hydroxy-8-(4-N,N-dimethylaminophenyl)-5,6,7,8-tetrahydroisoquinolin-3-yl)thio]acetamide** **(5).**

**Figure S11:** ^1^H NMR Spectrum of **2-[(7-Acetyl-4-cyano-1,6-dimethyl-6-hydroxy-8-(4-N,N-dimethylaminophenyl)-5,6,7,8-tetrahydroisoquinolin-3-yl)thio]acetamide** **(5).**

**Figure S12: ^13^**C NMR Spectrum **2-[(7-Acetyl-4-cyano-1,6-dimethyl-6-hydroxy-8-(4-N,N-dimethylaminophenyl)-5,6,7,8-tetrahydroisoquinolin-3-yl)thio]acetamide** **(5).**

**Figure S13**: IR Spectrum of **2-[(7-Acetyl-4-cyano-1,6-dimethyl-6-hydroxy-8-**

**(4-N,N-dimethylaminophenyl)-5,6,7,8-tetrahydroisoquinolin-3-yl)thio]acetonitrile** **(6)**.

**Figure S14:** ^1^H NMR Spectrum of **2-[(7-Acetyl-4-cyano-1,6-dimethyl-6-hydroxy-8-(4-N,N-dimethylaminophenyl)-5,6,7,8-tetrahydroisoquinolin-3-yl)thio]acetonitrile** **(6)**.

**Figure S15: ^13^**C NMR Spectrum **2-[(7-Acetyl-4-cyano-1,6-dimethyl-6-hydroxy-8-(4-N,N-dimethylaminophenyl)-5,6,7,8-tetrahydroisoquinolin-3-yl)thio]acetonitrile** **(6).**

**Figure S16**: IR Spectrum of **2-[(7-Acetyl-4-cyano-1,6-dimethyl-6-hydroxy-8-**

**(4-N,N-dimethylaminophenyl)-5,6,7,8-tetra-hydroisoquinolin-3-yl)thio]-*N*-phenylacetamide (7a).**

**Figure S17:** ^1^H NMR Spectrum o**f 2-[(7-Acetyl-4-cyano-1,6-dimethyl-6-hydroxy-8-(4-N,N-dimethylaminophenyl)-5,6,7,8-tetra-hydroisoquinolin-3-yl)thio]-*N*-phenylacetamide (7a).**

**Figure S18: ^13^**C NMR Spectrum of **2-[(7-Acetyl-4-cyano-1,6-dimethyl-6-hydroxy-8-(4-N,N-dimethylaminophenyl)-5,6,7,8-tetra-hydroisoquinolin-3-yl)thio]-*N*-phenylacetamide (7a).**

**Figure S19: IR Spectrum of 2-[(7-Acetyl-4-cyano-1,6-dimethyl-6-hydroxy-8-(4-N,N-dimethylaminophenyl)-5,6,7,8-tetrahydroisoquinolin-3-yl)thio]-*N*-(4-tolyl)acetamide** (7b).

**Figure S20:** ^1^H NMR Spectrum of **2-[(7-Acetyl-4-cyano-1,6-dimethyl-6-hydroxy-8-(4-N,N-dimethylaminophenyl)-5,6,7,8-tetrahydroisoquinolin-3-yl)thio]-*N*-(4-tolyl)acetamide** (**7b**).

**Figure S21: ^13^C NMR Spectrum of 2-[(7-Acetyl-4-cyano-1,6-dimethyl-6-hydroxy-8-(4-N,N-dimethylaminophenyl)-5,6,7,8-tetrahydroisoquinolin-3-yl)thio]-*N*-(4-tolyl)acetamide** (**7b**).

**Figure S22**: IR Spectrum of **2-[(7-Acetyl-4-cyano-1,6-dimethyl-6-hydroxy-8-(4-N,N-dimethylaminophenyl)-,5,6,7,8-tetrahydroisoquinolin-3-yl)thio]-*N*-(4-chlorophenyl)acetamide** (**7c**).

**Figure S23:** ^1^H NMR Spectrum of **2-[(7-Acetyl-4-cyano-1,6-dimethyl-6-hydroxy-8-(4-N,N-dimethylaminophenyl)-,5,6,7,8-tetrahydroisoquinolin-3-yl)thio]-*N*-(4-chlorophenyl)acetamide** (**7c**).

**Figure S24: ^13^**C NMR Spectrum of **2-[(7-Acetyl-4-cyano-1,6-dimethyl-6-hydroxy-8-(4-N,N-dimethylaminophenyl)-,5,6,7,8-tetrahydroisoquinolin-3-yl)thio]-*N*-(4-chlorophenyl)acetamide** (**7c**).

**Figure S25**: IR Spectrum of **2-[(7-Acetyl-4-cyano-1,6-dimethyl-6-hydroxy-8-(4-N,N-dimethylaminophenyl)-5,6,7,8-tetrahydroisoquinolin-3-yl)thio]-*N*-(4-acetylphenyl)acetamide** **(7d).**

**Figure S26:** ^1^H NMR Spectrum of **2-[(7-Acetyl-4-cyano-1,6-dimethyl-6-hydroxy-8-(4-N,N-dimethylaminophenyl)-5,6,7,8-tetrahydroisoquinolin-3-yl)thio]-*N*-(4-acetylphenyl)acetamide** **(7d)**

**Figure S27: ^13^**C NMR Spectrum **2-[(7-Acetyl-4-cyano-1,6-dimethyl-6-hydroxy-8-(4-N,N-dimethylaminophenyl)-5,6,7,8-tetrahydroisoquinolin-3-yl)thio]-*N*-(4-acetylphenyl)acetamide** **(7d)**

**Figure S28**: IR Spectrum of **2-[(7-Acetyl-4-cyano-1,6-dimethyl-6-hydroxy-8-(4-N,N-dimethylaminophenyl)-5,6,7,8-tetrahydroisoquinolin-3-yl)thio]-*N*** CH_3_**-(naphthalen-1-yl)acetamide (7e)**.

**Figure S29:** ^1^H NMR Spectrum of **2-[(7-Acetyl-4-cyano-1,6-dimethyl-6-hydroxy-8-**

**(4-N,N-dimethylaminophenyl)-5,6,7,8-tetrahydroisoquinolin-3-yl)thio]-*N*** CH_3_**-(naphthalen-1-yl)acetamide (7e).**

**Figure S30: ^13^**C NMR Spectrum of **2-[(7-Acetyl-4-cyano-1,6-dimethyl-6-hydroxy-8-(4-N,N-dimethylaminophenyl)-5,6,7,8-tetrahydroisoquinolin-3-yl)thio]-*N*** CH_3_**-(naphthalen-1-yl)acetamide (7e).**

**Figure S3**1: IR Spectrum of **7-Acetyl-1-amino-5,8-dimethyl-8-hydroxy-6-(4-N,N-dimethylaminophenyl)-*N*-phenyl-6,7,8,9-tetrahydrothieno[2,3-c]isoquinoline-2-carboxamide** **(8a).**

**Figure S32:** ^1^H NMR Spectrum of **7-Acetyl-1-amino-5,8-dimethyl-8-hydroxy-6-(4-N,N-dimethylaminophenyl)-*N*-phenyl-6,7,8,9-tetrahydrothieno[2,3-c]isoquinoline-2-carboxamide** **(8a).**

**Figure S33: ^13^**C NMR Spectrum of **7-Acetyl-1-amino-5,8-dimethyl-8-hydroxy-6-(4-N,N-dimethylaminophenyl)-*N*-phenyl-6,7,8,9-tetrahydrothieno[2,3-c]isoquinoline-2-carboxamide** **(8a).**

**Figure S34**: IR Spectrum of **7-Acetyl-1-amino-5,8-dimethyl-8-hydroxy-6-(4-N,N-dimethylaminophenyl)-*N*-(4-tolyl)-6,7,8,9-tetrahydrothieno[2,3-*c*]isoquinoline-2-carboxamide(8b).**

**Figure S35:** ^1^H NMR Spectrum of **7-Acetyl-1-amino-5,8-dimethyl-8-hydroxy-6-(4-N,N-dimethylaminophenyl)-*N*-(4-tolyl)-6,7,8,9-tetrahydrothieno[2,3-*c*]isoquinoline-2-carboxamide** **(8b).**

**Figure S36: ^13^**C NMR Spectrum of **7-Acetyl-1-amino-5,8-dimethyl-8-hydroxy-6-(4-N,N-dimethylaminophenyl)-*N*-(4-tolyl)-6,7,8,9-tetrahydrothieno[2,3-*c*]isoquinoline-2-carboxamide(8b).**

**Figure S37**: IR Spectrum of **7-Acetyl-1-amino-*N*-(4-chlorophenyl)-5,8-dimethyl-8-hydroxy-6-(4-N,N-dimethyl-aminophenyl)-6,7,8,9-tetrahydrothieno[2,3-*c*]isoquinoline-2-carboxamide** **(8c)**.

**Figure S38:** ^1^H NMR Spectrum of **7-Acetyl-1-amino-*N*-(4-chlorophenyl)5,8-dimethyl-8-hydroxy-6-**

**(4-N,N-dimethyl-aminophenyl)-6,7,8,9-tetrahydrothieno[2,3-*c*]isoquinoline-2-carboxamide** **(8c)**.

**Figure S39: ^13^**C NMR Spectrum of **7-Acetyl-1-amino-*N*-(4-chlorophenyl)-5,8-dimethyl-8-hydroxy-6-**

**(4-N,N-dimethyl-aminophenyl)-6,7,8,9-tetrahydrothieno[2,3-*c*]isoquinoline-2-carboxamide** **(8c)**.

**Figure S40**: IR Spectrum of 7**-Acetyl-*N*-(4-acetylphenyl)-1-amino-5,8-dimethyl-8-hydroxy-6-(4-N,N-dimethyl-aminophenyl)-6,7,8,9-tetrahydrothieno[2,3-*c*]isoquinoline-2-carboxamide (8d)**.

**Figure S41:** ^1^H NMR Spectrum of 7**-Acetyl-*N*-(4-acetylphenyl)-1-amino-5,8-dimethyl-8-hydroxy-6-(4-N,N-dimethyl-aminophenyl)-6,7,8,9-tetrahydrothieno[2,3-*c*]isoquinoline-2-carboxamide (8d)**.

**Figure S42: ^13^**C NMR Spectrum of 7**-Acetyl-*N*-(4-acetylphenyl)-1-amino-5,8-dimethyl-8-hydroxy-6-(4-N,N-dimethyl-aminophenyl)-6,7,8,9-tetrahydrothieno[2,3-*c*]isoquinoline-2-carboxamide (8d)**.

**Figure S43**: IR Spectrum of **7-Acetyl-1-amino-*N* (naphthalen-1-yl)-5,8-dimethyl-8-hydroxy-6-(4-N,N-dimethyl-aminophenyl)-6,7,8,9-tetrahydrothieno[2,3-*c*]isoquinoline-2-carboxamide** **(8e)**.

**Figure S44:** ^1^H NMR Spectrum o**7-Acetyl-1-amino-*N* (naphthalen-1-yl)-5,8-dimethyl-8-hydroxy-6-(4-N,N-dimethyl-aminophenyl)-6,7,8,9-tetrahydrothieno[2,3-*c*]isoquinoline-2-carboxamide** **(8e)**.

f

**Figure S45: ^13^**C NMR Spectrum of **7-Acetyl-1-amino-*N* (naphthalen-1-yl)-5,8-dimethyl-8-hydroxy-6**

**-(4-N,N-dimethyl-aminophenyl)-6,7,8,9-tetrahydrothieno[2,3-*c*]isoquinoline-2-carboxamide** **(8e)**.

**Table S1.** Raw date of toxicity and viability of compds 1,3,4,5 and 6 against MCF7.

| **ID** | **Conc.** | **O.D** | | | **Mean O.D** | **ST.E** | **Viability %** | **Toxicity %** | **IC_50_** |
| --- | --- | --- | --- | --- | --- | --- | --- | --- | --- |
|  | **µMol** |  |  |  |  |  |  |  | **µMol** |
| MCF7 | dilution | 0.815 | 0.81 | 0.823 | 0.816 | 0.003786 | 100 | 0 |  |
| doxorubicin | 25 | 0.021 | 0.024 | 0.018 | 0.021 | 0.001732 | 1.555500 | 98.45 | **0.053** |
|  | 12.5 | 0.023 | 0.034 | 0.028 | 0.028333 | 0.00318 | 2.542311 | 97.46 |  |
|  | 6.25 | 0.043 | 0.059 | 0.051 | 0.051 | 0.004619 | 6.5503501 | 93.45 |  |
|  | 3.125 | 0.081 | 0.092 | 0.088 | 0.087 | 0.003215 | 9.13000 | 90.87 |  |
|  | 1.562 | 0.118 | 0.119 | 0.107 | 0.114667 | 0.003844 | 19.771212 | 80.23 |  |
|  | 0.781 | 0.284 | 0.279 | 0.286 | 0.283 | 0.002082 | 26.44000 | 73.56 |  |
|  | 0.391 | 0.498 | 0.492 | 0.487 | 0.492333 | 0.00318 | 37.131112 | 62.87 |  |
|  | 0.195 | 0.654 | 0.651 | 0.663 | 0.656 | 0.003606 | 43.660011 | 56.34 |  |
|  | 0.0975 | 0.756 | 0.753 | 0.760 | 0.756 | 0.003200 | 47.34 | 52.667 |  |
|  | 0.0487 | 0.8498 | 0.850 | 0.848 | 0.849 | 0.00246 | 51.88 | 48.12 |  |
|  | 25 | 0.025 | 0.019 | 0.022 | 0.022 | 0.001732 | 2.6960784 | 97.303922 |  |
|  | 12.5 | 0.049 | 0.055 | 0.052 | 0.052 | 0.001732 | 6.372549 | 93.627451 |  |
|  | 6.25 | 0.111 | 0.103 | 0.106 | 0.106667 | 0.002333 | 13.071895 | 86.928105 | **1.857** |
| 1 | 3.125 | 0.194 | 0.187 | 0.198 | 0.193 | 0.003215 | 23.651961 | 76.348039 |  |
|  | 1.562 | 0.431 | 0.446 | 0.443 | 0.44 | 0.004583 | 53.921569 | 46.078431 |  |
|  | 0.781 | 0.621 | 0.624 | 0.627 | 0.624 | 0.001732 | 76.470588 | 23.529412 |  |
|  | 0.391 | 0.745 | 0.755 | 0.751 | 0.750 | 0.002906 | 91.952614 | 8.473856 |  |
|  | 0.195 | 0.817 | 0.809 | 0.811 | 0.812 | 0.002404 | 99.550654 | 0.4493464 |  |
|  | 0.0975 | 0.878 | 0.893 | 0.892 | 0.887 | 0.00345 | 99.67632 | 0.3245666 |  |
|  | 0.0487 | 0.923 | 0.913 | 0.932 | 0.915 | 0.920 | 99.725246 | 0.2744643 |  |
|  | 25 | 0.028 | 0.025 | 0.019 | 0.024 | 0.002646 | 2.9411765 | 97.058824 |  |
|  | 12.5 | 0.018 | 0.023 | 0.022 | 0.021 | 0.001528 | 2.5735294 | 97.426471 |  |
|  | 6.25 | 0.042 | 0.034 | 0.043 | 0.039667 | 0.002848 | 4.8611111 | 95.138889 |  |
| 3 | 3.125 | 0.064 | 0.075 | 0.076 | 0.071667 | 0.003844 | 8.7826797 | 91.21732 | **0.562** |
|  | 1.562 | 0.136 | 0.141 | 0.144 | 0.140333 | 0.002333 | 17.197712 | 82.802288 |  |
|  | 0.781 | 0.262 | 0.278 | 0.274 | 0.271333 | 0.004807 | 33.251634 | 66.748366 |  |
|  | 0.391 | 0.505 | 0.493 | 0.5 | 0.499333 | 0.00348 | 61.19281 | 38.80719 |  |
|  | 0.195 | 0.745 | 0.751 | 0.748 | 0.748 | 0.001732 | 91.666667 | 8.3333333 |  |
|  | 0.0975 | 0.792 | 0.782 | 0.7912 | 0.788 | 0.003455 | 92.876 | 7.124 |  |
|  | 0.0487 | 0.923 | 0.892 | 0.9123 | 0.909 | 0.001344 | 94.876 | 5.124 |  |
|  | 25 | 0.029 | 0.027 | 0.022 | 0.026 | 0.002082 | 3.1862745 | 96.813725 |  |
|  | 12.5 | 0.062 | 0.069 | 0.071 | 0.067333 | 0.002728 | 8.251634 | 91.748366 |  |
|  | 6.25 | 0.146 | 0.142 | 0.151 | 0.146333 | 0.002603 | 17.933007 | 82.066993 | **3.074** |
| 4 | 3.125 | 0.515 | 0.514 | 0.516 | 0.515 | 0.000577 | 63.112745 | 36.887255 |  |
|  | 1.562 | 0.621 | 0.628 | 0.626 | 0.625 | 0.002082 | 76.593137 | 23.406863 |  |
|  | 0.781 | 0.742 | 0.745 | 0.767 | 0.751333 | 0.007881 | 92.075163 | 7.9248366 |  |
|  | 0.391 | 0.818 | 0.812 | 0.819 | 0.816333 | 0.002186 | 100.04085 | 0.04085 |  |
|  | 0.195 | 0.819 | 0.817 | 0.81 | 0.815333 | 0.002728 | 99.918301 | 0.0816993 |  |
|  | 0.0975 | 0.927 | 0.921 | 0.912 | 0.93 | 0.002445 | 99.936676 | 0.064345 |  |
|  | 0.0487 | 0.941 | 0.993 | 0.978 | 0.9766 | 0.00244 | 99.938212 | 0.034667 |  |
|  | 25 | 0.027 | 0.021 | 0.02 | 0.022667 | 0.002186 | 2.7777778 | 97.222222 |  |
|  | 12.5 | 0.024 | 0.032 | 0.021 | 0.025667 | 0.003283 | 3.1454248 | 96.854575 |  |
|  | 6.25 | 0.055 | 0.051 | 0.043 | 0.049667 | 0.003528 | 6.0866013 | 93.913399 | **0.924** |
| 5 | 3.125 | 0.092 | 0.096 | 0.084 | 0.090667 | 0.003528 | 11.111111 | 88.888889 |  |
|  | 1.562 | 0.189 | 0.176 | 0.185 | 0.183333 | 0.003844 | 22.46732 | 77.53268 |  |
|  | 0.781 | 0.529 | 0.523 | 0.528 | 0.526667 | 0.001856 | 64.542484 | 35.457516 |  |
|  | 0.391 | 0.784 | 0.779 | 0.782 | 0.781667 | 0.001453 | 95.792484 | 4.2075163 |  |
|  | 0.195 | 0.834 | 0.846 | 0.838 | 0.839333 | 0.003528 | 102.85948 | -2.859477 |  |
|  | 0.0975 | 0.892 | 0.895 | 0.896 | 0.8945 | 0.00456 | 103.56655 | -3.54455 |  |
|  | 0.0487 | 0.923 | 0.956 | 0.9678 | 0.96578 | 0.00234 | 104.54433 | -4.56677 |  |
|  | 25 | 0.019 | 0.022 | 0.024 | 0.021667 | 0.001453 | 2.6552288 | 97.344771 |  |
|  | 12.5 | 0.023 | 0.025 | 0.022 | 0.023333 | 0.000882 | 2.8594771 | 97.140523 |  |
|  | 6.25 | 0.053 | 0.054 | 0.055 | 0.054 | 0.000577 | 6.6176471 | 93.382353 |  |
| 6 | 3.125 | 0.116 | 0.11 | 0.124 | 0.116667 | 0.004055 | 14.297386 | 85.702614 | **0.329** |
|  | 1.562 | 0.189 | 0.198 | 0.192 | 0.193 | 0.002646 | 23.651961 | 76.348039 |  |
|  | 0.781 | 0.234 | 0.241 | 0.242 | 0.239 | 0.002517 | 29.289216 | 70.710784 |  |
|  | 0.391 | 0.335 | 0.324 | 0.328 | 0.329 | 0.003215 | 40.318627 | 59.681373 |  |
|  | 0.195 | 0.507 | 0.5 | 0.505 | 0.504 | 0.002082 | 61.764706 | 38.235294 |  |
|  | 0.0975 | 0.591 | 0.596 | 0.582 | 0.589 | 0.00435 | 74.5543 | 25.5567 |  |
|  | 0.0487 | 0.674 | 0.635 | 0.634 | 0.647 | 0.00466 | 80.118 | 18.992 |  |

**Table S2.** Raw date of toxicity and viability of compounds **7a,7b,7c,7d,** and **7e** against **MCF7**.

| **ID** | **Conc.** | **O.D** | | | **Mean O.D** | **ST.E** | **Viability %** | **Toxicity %** | **IC_50_** |
| --- | --- | --- | --- | --- | --- | --- | --- | --- | --- |
|  | **µMol** |  |  |  |  |  |  |  | **µMol** |
| MCF7 | dilution | 0.815 | 0.81 | 0.823 | 0.816 | 0.003786 | 100 | 0 |  |
|  | 25 | 0.024 | 0.021 | 0.019 | 0.021333 | 0.001453 | 2.6143791 | 97.385621 |  |
|  | 12.5 | 0.064 | 0.061 | 0.072 | 0.065667 | 0.003283 | 8.0473856 | 91.952614 |  |
|  | 6.25 | 0.23 | 0.233 | 0.226 | 0.229667 | 0.002028 | 28.145425 | 71.854575 | **2.218** |
| 7a | 3.125 | 0.384 | 0.379 | 0.388 | 0.383667 | 0.002603 | 47.017974 | 52.982026 |  |
|  | 1.562 | 0.541 | 0.536 | 0.543 | 0.54 | 0.002082 | 66.176471 | 33.823529 |  |
|  | 0.781 | 0.631 | 0.624 | 0.627 | 0.627333 | 0.002028 | 76.879085 | 23.120915 |  |
|  | 0.391 | 0.745 | 0.755 | 0.751 | 0.750333 | 0.002906 | 91.952614 | 8.0473856 |  |
|  | 0.195 | 0.837 | 0.839 | 0.842 | 0.839333 | 0.001453 | 102.85948 | -2.859477 |  |
|  |  |  |  |  |  |  |  |  |  |
|  | 0.0975 | 0.895 | 0.894 | 0.896 | 0.895 | 0.00455 | 103.45566 | -3.455666 |  |
|  | 0.0487 | 0.945 | 0.9678 | 0.958 | 0.95666 | 0.0223445 | 105.76544 | -5.345662 |  |
|  | 25 | 0.028 | 0.025 | 0.031 | 0.028 | 0.001732 | 3.4313725 | 96.568627 |  |
|  | 12.5 | 0.027 | 0.034 | 0.022 | 0.027667 | 0.00348 | 3.3905229 | 96.609477 |  |
|  | 6.25 | 0.052 | 0.054 | 0.063 | 0.056333 | 0.003383 | 6.9035948 | 93.096405 | **0.474** |
| 7b | 3.125 | 0.111 | 0.115 | 0.106 | 0.110667 | 0.002603 | 13.562092 | 86.437908 |  |
|  | 1.562 | 0.146 | 0.141 | 0.144 | 0.143667 | 0.001453 | 17.606209 | 82.393791 |  |
|  | 0.781 | 0.162 | 0.178 | 0.274 | 0.204667 | 0.034973 | 25.081699 | 74.918301 |  |
|  | 0.391 | 0.485 | 0.499 | 0.494 | 0.492667 | 0.004096 | 60.375817 | 39.624183 |  |
|  | 0.195 | 0.745 | 0.751 | 0.748 | 0.748 | 0.001732 | 91.666667 | 8.3333333 |  |
|  | 0.0975 | 0.853 | 0.834 | 0.846 | 0.833 | 0.00494 | 93.77455 | 6.2344568 |  |
|  | 0.0487 | 0.921 | 0.923 | 926 | 0.9333 | 0.004566 | 95.4311 | 4.56888 |  |
|  | 25 | 0.029 | 0.023 | 0.027 | 0.026333 | 0.001764 | 3.2271242 | 96.772876 |  |
|  | 12.5 | 0.042 | 0.034 | 0.41 | 0.162 | 0.124022 | 19.852941 | 80.147059 |  |
|  | 6.25 | 0.109 | 0.114 | 0.111 | 0.111333 | 0.001453 | 13.643791 | 86.356209 | **1.491** |
| 7c | 3.125 | 0.275 | 0.284 | 0.286 | 0.281667 | 0.003383 | 34.517974 | 65.482026 |  |
|  | 1.562 | 0.522 | 0.512 | 0.516 | 0.516667 | 0.002906 | 63.316993 | 36.683007 |  |
|  | 0.781 | 0.742 | 0.745 | 0.757 | 0.748 | 0.004583 | 91.666667 | 8.3333333 |  |
|  | 0.391 | 0.819 | 0.819 | 0.814 | 0.817333 | 0.001667 | 100.1634 | 0.5333987 |  |
|  | 0.195 | 0.817 | 0.81 | 0.808 | 0.811667 | 0.002728 | 99.468954 | 0.1610458 |  |
|  | 0.0975 | 0.921 | 0.933 | 0.923 | 0.925 | 0.00778 | 100.57755 | -0.53355 |  |
|  | 0.0487 | 0.966 | 0.976 | 0.686 | 0.976 | 0.00345 | 100.43345 | -0.67765 |  |
|  | 25 | 0.028 | 0.03 | 0.022 | 0.026667 | 0.002404 | 3.2679739 | 96.732026 |  |
|  | 12.5 | 0.024 | 0.027 | 0.031 | 0.027333 | 0.002028 | 3.3496732 | 96.650327 |  |
|  |  |  |  |  |  |  |  |  |  |
|  | 6.25 | 0.045 | 0.048 | 0.053 | 0.048667 | 0.002333 | 5.9640523 | 94.035948 | **0.495** |
| 7d | 3.125 | 0.192 | 0.186 | 0.194 | 0.190667 | 0.002404 | 23.366013 | 76.633987 |  |
|  | 1.562 | 0.279 | 0.276 | 0.285 | 0.28 | 0.002646 | 34.313725 | 65.686275 |  |
|  | 0.781 | 0.389 | 0.385 | 0.388 | 0.387333 | 0.001202 | 47.46732 | 52.53268 |  |
|  | 0.391 | 0.584 | 0.579 | 0.582 | 0.581667 | 0.001453 | 71.28268 | 28.71732 |  |
|  | 0.195 | 0.778 | 0.766 | 0.779 | 0.774333 | 0.004177 | 94.893791 | 5.1062092 |  |
|  | 0.0975 | 0.812 | 0.8243 | 0.834 | 0.823 | 0.0016754 | 96.5544 | 3.5566 |  |
|  | 0.0487 | 0.953 | 0.9656 | 0.964 | 0.9 | 0.0034556 | 98.2255 | 1.8855 |  |
|  | 25 | 0.02 | 0.022 | 0.024 | 0.022 | 0.001155 | 2.6960784 | 97.303922 |  |
|  | 12.5 | 0.023 | 0.025 | 0.027 | 0.025 | 0.001155 | 3.0637255 | 96.936275 |  |
|  | 6.25 | 0.053 | 0.054 | 0.055 | 0.054 | 0.000577 | 6.6176471 | 93.382353 | **0.211** |
| 7e | 3.125 | 0.093 | 0.1 | 0.094 | 0.095667 | 0.002186 | 11.723856 | 88.276144 |  |
|  | 1.562 | 0.179 | 0.178 | 0.172 | 0.176333 | 0.002186 | 21.609477 | 78.390523 |  |
|  | 0.781 | 0.234 | 0.241 | 0.242 | 0.239 | 0.002517 | 29.289216 | 70.710784 |  |
|  | 0.391 | 0.31 | 0.314 | 0.318 | 0.314 | 0.002309 | 38.480392 | 61.519608 |  |
|  | 0.195 | 0.547 | 0.543 | 0.542 | 0.544 | 0.001528 | 66.666667 | 33.333333 |  |
|  | 0.0975 | 0.647 | 0.674 | 0.678 | 0.666 | 0.003456 | 73.554457 | 27.556667 |  |
|  | 0.0487 | 0.75 | 0.746 | 0.746 | 0.747 | 0.005868 | 79.234669 | 20.876446 |  |

**Tables S3.** Raw date of toxicity and viability of compounds **8a, 8b, 8c, 8d**, and **8e** against **MCF7**.

| **ID** | **Conc.**  **µMol** | **O.D** | | | **Mean O.D** | **ST.E** | **Viability %** | **Toxicity %** | **IC_50_**  **µMol** |
| --- | --- | --- | --- | --- | --- | --- | --- | --- | --- |
| MCF7 | dilution | 0.815 | 0.810 | 0.823 | 0.816 | 0.003786 | 100 | 0 |  |
| 8a | 25 | 0.025 | 0.018 | 0.020 | 0.021 | 0.002082 | 2.573529412 | 97.426470588 | **0.872** |
|  | 12.5 | 0.028 | 0.025 | 0.023 | 0.025333 | 0.001453 | 3.104575163 | 96.895424837 |  |
|  | 6.25 | 0.044 | 0.053 | 0.046 | 0.047667 | 0.002728 | 5.841503268 | 94.158496732 |  |
|  | 3.125 | 0.104 | 0.101 | 0.108 | 0.104333 | 0.002028 | 12.785947712 | 87.214052288 |  |
|  | 1.562 | 0.281 | 0.286 | 0.283 | 0.283333 | 0.001453 | 34.722222222 | 65.277777778 |  |
|  | 0.781 | 0.531 | 0.534 | 0.537 | 0.534 | 0.001732 | 65.44117647 | 34.558823529 |  |
|  | 0.391 | 0.745 | 0.755 | 0.751 | 0.750333 | 0.002906 | 91.95261438 | 8.047385621 |  |
|  | 0.195 | 0.817 | 0.819 | 0.822 | 0.819333 | 0.001453 | 100.40849673 | -0.408496732 |  |
|  | 0.0975 | 0.894 | 0.8934 | 0.893 | 0.8953 | 0.00345 | 100.57788655 | -0.53322455 |  |
|  | 0.0487 | 0.956 | 0.9465 | 0.945 | 0.949 | 0.00231 | 101.54432 | -1.566678 |  |
| 8b | 25 | 0.028 | 0.022 | 0.031 | 0.027 | 0.002646 | 3.308823529 | 96.691176471 | **3.800** |
|  | 12.5 | 0.097 | 0.093 | 0.102 | 0.097333 | 0.002603 | 11.928104575 | 88.071895425 |  |
|  | 6.25 | 0.322 | 0.326 | 0.328 | 0.325333 | 0.001764 | 39.869281046 | 60.130718954 |  |
|  | 3.125 | 0.614 | 0.615 | 0.616 | 0.615 | 0.000577 | 75.367647059 | 24.632352941 |  |
|  | 1.562 | 0.706 | 0.711 | 0.704 | 0.707 | 0.002082 | 86.642156863 | 13.357843137 |  |
|  | 0.781 | 0.792 | 0.800 | 0.804 | 0.798667 | 0.003528 | 97.87581699 | 2.124183007 |  |
|  | 0.391 | 0.824 | 0.811 | 0.818 | 0.817667 | 0.003756 | 100.20424837 | -0.204248366 |  |
|  | 0.195 | 0.813 | 0.811 | 0.819 | 0.814333 | 0.002404 | 99.79575163 | 0.204248366 |  |
|  | 0.0975 | 0.889 | 0.883 | 0.878 | 0.886 | 0.003423 | 99.8766 | 0.1234 |  |
|  | 0.0487 | 0.945 | 0.956 | 0.935 | 0.945 | 0.00233 | 99.9322 | 0.0678 |  |
| 8c | 25 | 0.029 | 0.027 | 0.019 | 0.025 | 0.003055 | 3.063725490 | 96.936274510 | **0.215** |
|  | 12.5 | 0.026 | 0.024 | 0.021 | 0.023667 | 0.001453 | 2.900326797 | 97.099673203 |  |
|  | 6.25 | 0.025 | 0.028 | 0.031 | 0.028 | 0.001732 | 3.431372549 | 96.568627451 |  |
|  | 3.125 | 0.027 | 0.024 | 0.036 | 0.029 | 0.003606 | 3.553921569 | 96.446078431 |  |
|  | 1.562 | 0.114 | 0.120 | 0.116 | 0.116667 | 0.001764 | 14.297385621 | 85.702614379 |  |
|  | 0.781 | 0.242 | 0.245 | 0.247 | 0.244667 | 0.001453 | 29.98366013 | 70.016339869 |  |
|  | 0.391 | 0.324 | 0.320 | 0.329 | 0.324333 | 0.002603 | 39.74673203 | 60.253267974 |  |
|  | 0.195 | 0.492 | 0.487 | 0.481 | 0.486667 | 0.00318 | 59.64052288 | 40.359477124 |  |
|  | 0.0975 | 0.564 | 0.546 | 0.567 | 0.559 | 0.00342 | 69.5434 | 30.4566 |  |
|  | 0.0487 | 0.646 | 0.678 | 0.648 | 0.657 | 0.00124 | 73.3223 | 26.67777 |  |
| 8d | 25 | 0.022 | 0.018 | 0.020 | 0.02 | 0.001155 | 2.450980392 | 97.549019608 | **0.117** |
|  | 12.5 | 0.024 | 0.022 | 0.026 | 0.024 | 0.001155 | 2.941176471 | 97.058823529 |  |
|  | 6.25 | 0.025 | 0.033 | 0.030 | 0.029333 | 0.002333 | 3.594771242 | 96.405228758 |  |
|  | 3.125 | 0.042 | 0.046 | 0.044 | 0.044 | 0.001155 | 5.392156863 | 94.607843137 |  |
|  | 1.562 | 0.050 | 0.056 | 0.045 | 0.050333 | 0.00318 | 6.168300654 | 93.831699346 |  |
|  | 0.781 | 0.097 | 0.093 | 0.089 | 0.093 | 0.002309 | 11.39705882 | 88.602941176 |  |
|  | 0.391 | 0.284 | 0.279 | 0.282 | 0.281667 | 0.001453 | 34.51797386 | 65.482026144 |  |
|  | 0.195 | 0.378 | 0.366 | 0.379 | 0.374333 | 0.004177 | 45.87418301 | 54.125816993 |  |
|  | 0.0975 | 0.456 | 0.425 | 0.467 | 0.449 | 0.002343 | 54.4424 | 45.6686 |  |
|  | 0.0487 | 0.578 | 0.567 | 0.578 | 0.574 | 0.00235 | 78.2125 | 21.78754 |  |
| 8e | 25 | 0.027 | 0.020 | 0.024 | 0.023667 | 0.002028 | 2.900326797 | 97.099673203 | **0.461** |
|  | 12.5 | 0.023 | 0.025 | 0.028 | 0.025333 | 0.001453 | 3.104575163 | 96.895424837 |  |
|  | 6.25 | 0.053 | 0.054 | 0.055 | 0.054 | 0.000577 | 6.617647059 | 93.382352941 |  |
|  | 3.125 | 0.117 | 0.114 | 0.121 | 0.117333 | 0.002028 | 14.379084967 | 85.620915033 |  |
|  | 1.562 | 0.289 | 0.298 | 0.292 | 0.293 | 0.002646 | 35.906862745 | 64.093137255 |  |
|  | 0.781 | 0.334 | 0.341 | 0.342 | 0.339 | 0.002517 | 41.54411765 | 58.455882353 |  |
|  | 0.391 | 0.645 | 0.644 | 0.648 | 0.645667 | 0.001202 | 79.12581699 | 20.874183007 |  |
|  | 0.195 | 0.779 | 0.780 | 0.772 | 0.777 | 0.002517 | 95.22058824 | 4.779411765 |  |
|  | 0.0975 | 0.823 | 0.845 | 0.823 | 0.83033 | 0.003239 | 97.37795 | 2.67546788 |  |
|  | 0.0487 | 0.923 | 0.945 | 0.914 | 0.92733 | 0.002349 | 98.423 | 1.5778654 |  |

##

## Table S4. Raw date of toxicity and viability of compounds 1,3,4,5, and 6 against A549.

| ID | Conc. | O.D | | | Mean O.D | ST.E | Viability % | Toxicity % | IC_50_ |
| --- | --- | --- | --- | --- | --- | --- | --- | --- | --- |
|  | **µMol** |  |  |  |  |  |  |  | **µMol** |
| A549 | dilution | 0.842 | 0.855 | 0.838 | 0.845 | 0.005132 | 100 | 0 |  |
| Douxrbicin aganist A549 | 25 | 0.021 | 0.024 | 0.021 | 0.022 | 0.001 | 1.10011044 | 98.9 | 0.218 |
|  | 12.5 | 0.073 | 0.084 | 0.083 | 0.08 | 0.003512 | 2.10001433 | 97.9 |  |
|  | 6.25 | 0.183 | 0.191 | 0.196 | 0.19 | 0.003786 | 4.65133122 | 95.35 |  |
|  | 3.125 | 0.489 | 0.487 | 0.478 | 0.484667 | 0.003383 | 6.44046566 | 93.56 |  |
|  | 1.562 | 0.668 | 0.679 | 0.677 | 0.674667 | 0.003383 | 10.1609787 | 89.84 |  |
|  | 0.781 | 0.714 | 0.707 | 0.716 | 0.712333 | 0.002728 | 20.260977 | 79.34 |  |
|  | 0.391 | 0.848 | 0.842 | 0.837 | 0.842333 | 0.00318 | 37.551247 | 62.45 |  |
|  | 0.195 | 0.984 | 0.991 | 0.973 | 0.982667 | 0.005239 | 63.670356 | 36.33 |  |
|  | 0.0975 | 0.99 | 0.998 | 0.989 | 0.99 | 0.00673 | 73.2 | 27.8 |  |
|  | 0.0487 | 0.996 | 0.997 | 0.995 | 0.99 | 0.00734 | 80 | 20 |  |
|  | 25 | 0.023 | 0.025 | 0.032 | 0.026667 | 0.002728 | 3.1558185 | 96.844181 |  |
|  | 12.5 | 0.078 | 0.076 | 0.08 | 0.078 | 0.001155 | 9.2307692 | 90.769231 |  |
| 1 | 6.25 | 0.2 | 0.205 | 0.198 | 0.201 | 0.002082 | 23.786982 | 76.213018 | 2.219 |
|  | 3.125 | 0.342 | 0.34 | 0.338 | 0.34 | 0.001155 | 40.236686 | 59.763314 |  |
|  | 1.562 | 0.55 | 0.545 | 0.553 | 0.549333 | 0.002333 | 65.009862 | 34.990138 |  |
|  | 0.781 | 0.663 | 0.66 | 0.658 | 0.660333 | 0.001453 | 78.145957 | 21.854043 |  |
|  | 0.391 | 0.782 | 0.776 | 0.777 | 0.778333 | 0.001856 | 92.110454 | 7.8895464 |  |
|  | 0.195 | 0.839 | 0.849 | 0.847 | 0.845 | 0.002055 | 97 | 3 |  |
|  | 0.0975 | 0.910 | 0.900 | 0.905 | 0.907 | 0.00377 | 98.2 | 2.8 |  |
|  | 0.0487 | 0.97 | 0.975 | 0.973 | 0.974 | 0.0043 | 99 | 1 |  |
|  | 25 | 0.03 | 0.026 | 0.034 | 0.03 | 0.002309 | 3.5502959 | 96.449704 |  |
|  | 12.5 | 0.09 | 0.086 | 0.092 | 0.089333 | 0.001764 | 10.571992 | 89.428008 |  |
| 3 | 6.25 | 0.244 | 0.25 | 0.248 | 0.247333 | 0.001764 | 29.270217 | 70.729783 | 2.469 |
|  | 3.125 | 0.401 | 0.389 | 0.397 | 0.395667 | 0.003528 | 46.824458 | 53.175542 |  |
|  | 1.562 | 0.52 | 0.522 | 0.525 | 0.522333 | 0.001453 | 61.814596 | 38.185404 |  |
|  | 0.781 | 0.644 | 0.645 | 0.648 | 0.645667 | 0.001202 | 76.410256 | 23.589744 |  |
|  | 0.391 | 0.799 | 0.805 | 0.802 | 0.802 | 0.001732 | 94.911243 | 5.0887574 |  |
|  | 0.195 | 0.837 | 0.838 | 0.84 | 0.838333 | 0.001882 | 99.211045 | 0.7889546 |  |
|  | 0.0975 | 0.897 | 0.895 | 0.893 | 0.895 | 0.00202 | 99.50 | 0.50 |  |
|  | 0.0487 | 0.900 | 0.92 | 0.94 | 0.920 | 0.00240 | 99.700 | 0.30 |  |
|  | 25 | 0.023 | 0.026 | 0.02 | 0.023 | 0.001732 | 2.7218935 | 97.278107 |  |
|  | 12.5 | 0.037 | 0.034 | 0.032 | 0.034333 | 0.001453 | 4.0631164 | 95.936884 |  |
| 4 | 6.25 | 0.088 | 0.092 | 0.094 | 0.091333 | 0.001764 | 10.808679 | 89.191321 | 0.918 |
|  | 3.125 | 0.243 | 0.247 | 0.246 | 0.245333 | 0.001202 | 29.033531 | 70.966469 |  |
|  | 1.562 | 0.32 | 0.322 | 0.318 | 0.32 | 0.001155 | 37.869822 | 62.130178 |  |
|  | 0.781 | 0.57 | 0.572 | 0.568 | 0.57 | 0.001155 | 67.455621 | 32.544379 |  |
|  | 0.391 | 0.661 | 0.668 | 0.67 | 0.666333 | 0.002728 | 78.856016 | 21.143984 |  |
|  | 0.195 | 0.807 | 0.816 | 0.812 | 0.811667 | 0.002803 | 96.055227 | 3.9447732 |  |
|  | 0.0975 | 0.820 | 0.843 | 0.876 | 0.843012 | 0.003955 | 98 | 2 |  |
|  | 0.0487 | 0.900 | 0.893 | 0.884 | 0.89688 | 0.004566 | 98.2 | 1.8 |  |
|  | 25 | 0.03 | 0.034 | 0.028 | 0.030667 | 0.001764 | 3.6291913 | 96.370809 |  |
|  | 12.5 | 0.067 | 0.072 | 0.074 | 0.071 | 0.002082 | 8.4023669 | 91.597633 |  |
| 5 | 6.25 | 0.144 | 0.139 | 0.141 | 0.141333 | 0.001453 | 16.725838 | 83.274162 | 1.247 |
|  | 3.125 | 0.258 | 0.259 | 0.252 | 0.256333 | 0.002186 | 30.335306 | 69.664694 |  |
|  | 1.562 | 0.388 | 0.386 | 0.39 | 0.388 | 0.001155 | 45.91716 | 54.08284 |  |
|  | 0.781 | 0.533 | 0.53 | 0.535 | 0.532667 | 0.001453 | 63.037475 | 36.962525 |  |
|  | 0.391 | 0.768 | 0.763 | 0.76 | 0.763667 | 0.002333 | 90.374753 | 9.6252465 |  |
|  | 0.195 | 0.848 | 0.84 | 0.839 | 0.842333 | 0.002848 | 99.684418 | 0.3155819 |  |
|  | 0.0975 | 0.8933 | 0.904 | 0.912 | 0.9003 | 0.00322 | 99.87 | 0.13 |  |
|  | 0.0487 | 0.9100 | 0.9232 | 0.921 | 0.91800 | 0.0012 | 100 | 0 |  |
|  | 25 | 0.032 | 0.028 | 0.034 | 0.031333 | 0.001764 | 3.7080868 | 96.291913 |  |
|  | 12.5 | 0.097 | 0.099 | 0.095 | 0.097 | 0.001155 | 11.47929 | 88.52071 |  |
| 6 | 6.25 | 0.211 | 0.214 | 0.216 | 0.213667 | 0.001453 | 25.285996 | 74.714004 | 3.736 |
|  | 3.125 | 0.478 | 0.472 | 0.474 | 0.474667 | 0.001764 | 56.17357 | 43.82643 |  |
|  | 1.562 | 0.591 | 0.597 | 0.594 | 0.594 | 0.001732 | 70.295858 | 29.704142 |  |
|  | 0.781 | 0.711 | 0.718 | 0.714 | 0.714333 | 0.002028 | 84.536489 | 15.463511 |  |
|  | 0.391 | 0.833 | 0.835 | 0.83 | 0.832667 | 0.001453 | 98.540434 | 1.4595661 |  |
|  | 0.195 | 0.839 | 0.836 | 0.844 | 0.839667 | 0.002333 | 99.368836 | 0.6311637 |  |
|  |  |  |  |  |  |  |  |  |  |
|  | 0.0975 | 0.8588 | 0.867 | 0.8723 | 0.8643 | 0.00321 | 99.5 | 0.50 |  |
|  |  |  |  |  |  | 0.00356 |  | 0.4 |  |
|  | 0.0487 | 0.899 | 0.896 | 0.897 | 0.897 |  | 99.7 |  |  |

**Table S5.** Raw date of toxicity and viability of compounds **7a,7b,7c,7d**, and **7e** against **A549**.

| ID | Conc. | O.D | | | Mean O.D | ST.E | Viability % | **Toxicity %** | **IC_50_** |
| --- | --- | --- | --- | --- | --- | --- | --- | --- | --- |
|  | **µMol** |  |  |  |  |  |  |  | **µMol** |
| A549 | dilution | 0.842 | 0.855 | 0.838 | 0.845 | 0.005132 | 100 | 0 |  |
|  | 25 | 0.033 | 0.027 | 0.03 | 0.03 | 0.001732 | 3.0241935 | 96.975806 |  |
|  | 12.5 | 0.087 | 0.082 | 0.089 | 0.086 | 0.002082 | 8.6693548 | 91.330645 |  |
| 7a | 6.25 | 0.2 | 0.194 | 0.198 | 0.1973 | 0.001764 | 19.892473 | 80.107527 | 1.586 |
|  | 3.125 | 0.463 | 0.46 | 0.466 | 0.463 | 0.001732 | 46.673387 | 53.326613 |  |
|  | 1.562 | 0.584 | 0.58 | 0.582 | 0.582 | 0.001155 | 58.669355 | 41.330645 |  |
|  |  |  |  |  |  |  |  |  |  |
|  | 0.781 | 0.699 | 0.691 | 0.695 | 0.695 | 0.002309 | 70.060484 | 29.939516 |  |
|  | 0.391 | 0.855 | 0.851 | 0.856 | 0.854 | 0.001528 | 86.08871 | 13.91129 |  |
|  | 0.195 | 0.989 | 0.992 | 0.99 | 0.9903 | 0.000882 | 99.831989 | 0.1680108 |  |
|  | 0.0975 | 0.995 | 0.993 | 0.996 | 0.99466 | 0.001234 | 99.86545 | 0.134556 |  |
|  | 0.04875 | 0.998 | 0.997 | 0.998 | 0.99766 | 0.001453 | 99.90118 | 0.09882 |  |
|  | 25 | 0.033 | 0.03 | 0.028 | 0.030333 | 0.001453 | 3.5897436 | 96.410256 |  |
|  | 12.5 | 0.05 | 0.054 | 0.052 | 0.052 | 0.001155 | 6.1538462 | 93.846154 |  |
| 7b | 6.25 | 0.112 | 0.118 | 0.115 | 0.115 | 0.001732 | 13.609467 | 86.390533 | 0.987 |
|  | 3.125 | 0.234 | 0.23 | 0.236 | 0.233333 | 0.001764 | 27.613412 | 72.386588 |  |
|  | 1.562 | 0.39 | 0.393 | 0.388 | 0.390333 | 0.001453 | 46.193294 | 53.806706 |  |
|  | 0.781 | 0.542 | 0.54 | 0.545 | 0.542333 | 0.001453 | 64.18146 | 35.81854 |  |
|  | 0.391 | 0.722 | 0.728 | 0.725 | 0.725 | 0.001732 | 85.798817 | 14.201183 |  |
|  | 0.195 | 0.846 | 0.836 | 0.84 | 0.840667 | 0.002906 | 99.487179 | 0.5128205 |  |
|  | 0.0975 | 0.889 | 0.888 | 0.89 | 0.889 | 0.00345 | 99.6534 | 0.3466567 |  |
|  | 0.04875 | 0.956 | 0.976 | 0.959 | 0.974 | 0.012545 | 99.7534 | 0.246666 |  |
|  | 25 | 0.018 | 0.022 | 0.025 | 0.021667 | 0.002028 | 2.5641026 | 97.435897 |  |
|  | 12.5 | 0.035 | 0.036 | 0.032 | 0.034333 | 0.001202 | 4.0631164 | 95.936884 |  |
| 7c | 6.25 | 0.077 | 0.082 | 0.08 | 0.079667 | 0.001453 | 9.4280079 | 90.571992 | 0.496 |
|  | 3.125 | 0.19 | 0.193 | 0.195 | 0.192667 | 0.001453 | 22.800789 | 77.199211 |  |
|  | 1.562 | 0.266 | 0.271 | 0.273 | 0.27 | 0.002082 | 31.952663 | 68.047337 |  |
|  | 0.781 | 0.41 | 0.408 | 0.412 | 0.41 | 0.001155 | 48.52071 | 51.47929 |  |
|  | 0.391 | 0.56 | 0.566 | 0.562 | 0.562667 | 0.001764 | 66.587771 | 33.412229 |  |
|  | 0.195 | 0.789 | 0.793 | 0.796 | 0.792667 | 0.002028 | 93.806706 | 6.1932939 |  |
|  | 0.0975 | 0.8332 | 0.845 | 0.846 | 0.8414 | 0.002135 | 95.3422 | 4.6578766 |  |
|  | 0.04875 | 0.956 | 0.964 | 0.958 | 0.9593 | 0.001847 | 96.54323 | 3.456777 |  |
|  | 25 | 0.032 | 0.028 | 0.026 | 0.028667 | 0.001764 | 3.3925049 | 96.607495 |  |
|  | 12.5 | 0.056 | 0.062 | 0.06 | 0.059333 | 0.001764 | 7.0216963 | 92.978304 |  |
| 7d | 6.25 | 0.112 | 0.109 | 0.114 | 0.111667 | 0.001453 | 13.21499 | 86.78501 | 0.446 |
|  | 3.125 | 0.19 | 0.198 | 0.193 | 0.193667 | 0.002333 | 22.919132 | 77.080868 |  |
|  | 1.562 | 0.247 | 0.249 | 0.253 | 0.249667 | 0.001764 | 29.546351 | 70.453649 |  |
|  | 0.781 | 0.389 | 0.393 | 0.387 | 0.389667 | 0.001764 | 46.114398 | 53.885602 |  |
|  | 0.391 | 0.53 | 0.538 | 0.534 | 0.534 | 0.002309 | 63.195266 | 36.804734 |  |
|  | 0.195 | 0.81 | 0.815 | 0.816 | 0.813667 | 0.001856 | 96.291913 | 3.7080868 |  |
|  | 0.0975 | 0.901 | 0.91 | 0.906 | 0.90566 | 0.001456 | 97.5431 | 2.4569 |  |
|  | 0.04875 | 0.978 | 0.976 | 0.965 | 0.973 | 0.001345 | 98.01817 | 1.9818378 |  |
|  | 25 | 0.02 | 0.024 | 0.018 | 0.020667 | 0.001764 | 2.4457594 | 97.554241 |  |
|  | 12.5 | 0.023 | 0.016 | 0.019 | 0.019333 | 0.002028 | 2.2879684 | 97.712032 |  |
| 7e | 6.25 | 0.013 | 0.028 | 0.022 | 0.021 | 0.004359 | 2.4852071 | 97.514793 | 0.155 |
|  | 3.125 | 0.042 | 0.038 | 0.044 | 0.041333 | 0.001764 | 4.8915187 | 95.108481 |  |
|  | 1.562 | 0.09 | 0.086 | 0.092 | 0.089333 | 0.001764 | 10.571992 | 89.428008 |  |
|  | 0.781 | 0.15 | 0.158 | 0.157 | 0.155 | 0.002517 | 18.343195 | 81.656805 |  |
|  | 0.391 | 0.29 | 0.285 | 0.288 | 0.287667 | 0.001453 | 34.043393 | 65.956607 |  |
|  | 0.195 | 0.52 | 0.524 | 0.518 | 0.520667 | 0.001764 | 61.617357 | 38.382643 |  |
|  | 0.0975 | 0.634 | 0.621 | 0.623 | 0.626 | 0.00168 | 70.5142 | 29.4858 |  |
|  | 0.04875 | 0.732 | 0.734 | 0.745 | 0.737 | 0.001895 | 76.8061 | 23.193943 |  |

**Tables S6.** Raw date of toxicity and viability of compounds **8a, 8b, 8c, 8d**, and **8e** against **A549**.

| ID | Conc.  **µMol** | O.D | | | Mean O.D | ST.E | Viability % | Toxicity % | IC_50_  **µMol** |
| --- | --- | --- | --- | --- | --- | --- | --- | --- | --- |
| A549 | dilution | 0.842 | 0.855 | 0.838 | 0.845 | 0.005132 | 100 | 0 |  |
| 8a | 25 | 0.017 | 0.015 | 0.020 | 0.017333 | 0.001453 | 2.051282051 | 97.948717949 | 1.045 |
|  | 12.5 | 0.030 | 0.026 | 0.028 | 0.028 | 0.001155 | 3.313609467 | 96.686390533 |  |
|  | 6.25 | 0.099 | 0.102 | 0.097 | 0.099333 | 0.001453 | 11.755424063 | 88.244575937 |  |
|  | 3.125 | 0.211 | 0.217 | 0.214 | 0.214 | 0.001732 | 25.325443787 | 74.674556213 |  |
|  | 1.562 | 0.392 | 0.390 | 0.395 | 0.392333 | 0.001453 | 46.429980276 | 53.570019724 |  |
|  | 0.781 | 0.510 | 0.508 | 0.513 | 0.510333 | 0.001453 | 60.394477318 | 39.605522682 |  |
|  | 0.391 | 0.750 | 0.755 | 0.757 | 0.754 | 0.002082 | 89.23076923 | 10.769230769 |  |
|  | 0.195 | 0.809 | 0.813 | 0.806 | 0.809333 | 0.002028 | 95.77909270 | 4.220907298 |  |
|  | 0.0975 | 0.893 | 0.874 | 0.879 | 0.88213 | 0.002145 | 96.3211224 | 3.6788776 |  |
|  | 0.04875 | 0.956 | 0.965 | 0.976 | 0.9656 | 0.002095 | 96.8654434 | 3.1345566 |  |
| 8b | 25 | 0.030 | 0.034 | 0.036 | 0.033333 | 0.001764 | 3.944773176 | 96.055226824 | 0.527 |
|  | 12.5 | 0.050 | 0.067 | 0.054 | 0.057 | 0.005132 | 6.745562130 | 93.254437870 |  |
|  | 6.25 | 0.133 | 0.136 | 0.129 | 0.132667 | 0.002028 | 15.700197239 | 84.299802761 |  |
|  | 3.125 | 0.250 | 0.245 | 0.248 | 0.247667 | 0.001453 | 29.309664694 | 70.690335306 |  |
|  | 1.562 | 0.320 | 0.326 | 0.332 | 0.326 | 0.003464 | 38.579881657 | 61.420118343 |  |
|  | 0.781 | 0.408 | 0.407 | 0.403 | 0.406 | 0.001528 | 48.047337278 | 51.952662722 |  |
|  | 0.391 | 0.666 | 0.664 | 0.662 | 0.664 | 0.001155 | 78.57988166 | 21.420118343 |  |
|  | 0.195 | 0.731 | 0.736 | 0.749 | 0.738667 | 0.005364 | 87.41617357 | 12.583826430 |  |
|  | 0.0975 | 0.798 | 0.792 | 0.79 | 0.7933 | 0.003212 | 89.51415 | 10.4858548 |  |
|  | 0.04875 | 0.854 | 0.842 | 0.844 | 0.84666 | 0.002145 | 91.0766 | 8.9293384 |  |
| 8c | 25 | 0.020 | 0.030 | 0.026 | 0.025333 | 0.002906 | 2.998027613 | 97.001972387 | 0.332 |
|  | 12.5 | 0.023 | 0.027 | 0.034 | 0.028 | 0.003215 | 3.313609467 | 96.686390533 |  |
|  | 6.25 | 0.088 | 0.090 | 0.092 | 0.09 | 0.001155 | 10.650887574 | 89.349112426 |  |
|  | 3.125 | 0.134 | 0.141 | 0.126 | 0.133667 | 0.004333 | 15.818540434 | 84.181459566 |  |
|  | 1.562 | 0.210 | 0.200 | 0.205 | 0.205 | 0.002887 | 24.260355030 | 75.739644970 |  |
|  | 0.781 | 0.303 | 0.290 | 0.293 | 0.295333 | 0.00393 | 34.950690335 | 65.049309665 |  |
|  | 0.391 | 0.443 | 0.447 | 0.450 | 0.446667 | 0.002028 | 52.85996055 | 47.140039448 |  |
|  | 0.195 | 0.690 | 0.687 | 0.683 | 0.686667 | 0.002028 | 81.26232742 | 18.737672584 |  |
|  | 0.0975 | 0.734 | 0.735 | 0.743 | 0.73733 | 0.002148 | 85.74305 | 14.2569595 |  |
|  | 0.04875 | 0.823 | 0.843 | 0.831 | 0.83233 | 0.0020459 | 89.7605152 | 10.2394848 |  |
| 8d | 25 | 0.022 | 0.030 | 0.033 | 0.028333 | 0.003283 | 3.353057199 | 96.646942801 | 0.515 |
|  | 12.5 | 0.027 | 0.029 | 0.035 | 0.030333 | 0.002404 | 3.589743590 | 96.410256410 |  |
|  | 6.25 | 0.097 | 0.104 | 0.093 | 0.098 | 0.003215 | 11.597633136 | 88.402366864 |  |
|  | 3.125 | 0.144 | 0.149 | 0.146 | 0.146333 | 0.001453 | 17.317554241 | 82.682445759 |  |
|  | 1.562 | 0.265 | 0.269 | 0.267 | 0.267 | 0.001155 | 31.597633136 | 68.402366864 |  |
|  | 0.781 | 0.390 | 0.396 | 0.394 | 0.393333 | 0.001764 | 46.548323471 | 53.451676529 |  |
|  | 0.391 | 0.477 | 0.472 | 0.474 | 0.474333 | 0.001453 | 56.13412229 | 43.865877712 |  |
|  | 0.195 | 0.686 | 0.680 | 0.683 | 0.683 | 0.001732 | 80.82840237 | 19.171597633 |  |
|  | 0.0975 | 0.721 | 0.723 | 0.731 | 0.725 | 0.001456 | 84.60513456 | 15.39493982 |  |
|  | 0.04875 | 0.832 | 0.821 | 0.834 | 0.829 | 0.001234 | 86.5050687 | 13.49494848 |  |
| 8e | 25 | 0.022 | 0.017 | 0.020 | 0.019667 | 0.001453 | 2.327416174 | 97.672583826 | 1.329 |
|  | 12.5 | 0.030 | 0.028 | 0.022 | 0.026667 | 0.002404 | 3.155818540 | 96.844181460 |  |
|  | 6.25 | 0.098 | 0.095 | 0.100 | 0.097667 | 0.001453 | 11.558185404 | 88.441814596 |  |
|  | 3.125 | 0.266 | 0.262 | 0.260 | 0.262667 | 0.001764 | 31.084812623 | 68.915187377 |  |
|  | 1.562 | 0.478 | 0.472 | 0.470 | 0.473333 | 0.002404 | 56.015779093 | 43.984220907 |  |
|  | 0.781 | 0.540 | 0.538 | 0.542 | 0.540 | 0.001155 | 63.905325444 | 36.094674556 |  |
|  | 0.391 | 0.776 | 0.780 | 0.773 | 0.776333 | 0.002028 | 91.87376726 | 8.126232742 |  |
|  | 0.195 | 0.833 | 0.840 | 0.846 | 0.839667 | 0.003756 | 99.36883629 | 0.631163708 |  |
|  | 0.0975 | 0.893 | 0.892 | 0.887 | 0.89066 | 0.003459 | 99.65436 | 0.34564356 |  |
|  | 0.04875 | 0.923 | 0.931 | 0.924 | 0.926 | 0.00432 | 99.7606 | 0.2394855 |  |

**Table S6a: cytotoxicity of compound 7e, 8d on WI38 cell line**

|  |  | |  |  |  |  |  |  |  |  |  |  |
| --- | --- | --- | --- | --- | --- | --- | --- | --- | --- | --- | --- | --- |
|  |  | | |  |  |  |  | |  |  |  |  |
|  |  |  |  |  |  |  |  |  |  |  |  |  |
|  |  |  |  |  |  |  |  |  |  |  |  |  |
|  |  |  |  |  |  |  |  |  |  |  |  |  |
|  | **Blank** | **CC** | **Sample No. 7e/WI38** | | | | | **Sample No. 8d/WI38** | | | | |
|  | **1** | **2** | **3** | **4** | **5** | **6** | **7** | **8** | **9** | **10** | **11** | **12** |
| A | B | C | 25uM | 12.5uM | 6.25uM | 3.25uM | 1.55uM | 25uM | 12.5uM | 6.25uM | 3.25uM | 1.55uM |
| B | B | C | 25uM | 12.5uM | 6.25uM | 3.25uM | 1.55uM | 25uM | 12.5uM | 6.25uM | 3.25uM | 1.55uM |
| C | B | C | 25uM | 12.5uM | 6.25uM | 3.25uM | 1.55uM | 25uM | 12.5uM | 6.25uM | 3.25uM | 1.55uM |
| ROBONIK P2000 Eia reader | | | |  |  |  |  |  |  |  |  |  |
| Wave length: 450 nm | | | |  |  |  |  |  |  |  |  |  |
| Reference: 630 nm | | | |  |  |  |  |  |  |  |  |  |
|  | **1** | **2** | **3** | **4** | **5** | **6** | **7** | **8** | **9** | **10** | **11** | **12** |
|  |  |  |  |  |  |  |  |  |  |  |  |  |
| A | 0.001 | 0.495 | 0.216 | 0.284 | 0.354 | 0.416 | 0.477 | 0.231 | 0.311 | 0.379 | 0.428 | 0.487 |
| B | 0.003 | 0.509 | 0.222 | 0.292 | 0.349 | 0.422 | 0.481 | 0.229 | 0.305 | 0.364 | 0.439 | 0.484 |
| C | 0.001 | 0.483 | 0.231 | 0.307 | 0.341 | 0.405 | 0.464 | 0.237 | 0.318 | 0.357 | 0.416 | 0.467 |
| mean | 0.002 | 0.496 | 0.223 | 0.29433 | 0.348 | 0.41433 | 0.474 | 0.2323 | 0.31133 | 0.366667 | 0.42767 | 0.47933 |
| % viability |  |  | 44.9899 | 59.3813 | 70.208 | 83.5911 | 95.6288 | 46.873 | 62.811 | 73.97445 | 86.2811 | 96.7048 |
| 7e/WI38 | | 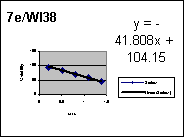 |  |  |  |  |  | 8d/WI38 |  | 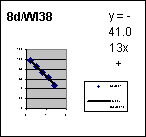 |  |  |
| 1.4 | 44.99 |  |  |  |  |  |  | 1.3979 | 46.8729 |  |  |  |
| 1.1 | 59.38 |  |  |  |  |  |  | 1.0969 | 62.811 |  |  |  |
| 0.8 | 70.21 |  |  |  |  |  |  | 0.7959 | 73.9744 |  |  |  |
| 0.51 | 83.59 |  |  |  |  |  |  | 0.5119 | 86.2811 |  |  |  |
| 0.19 | 95.63 |  |  |  |  |  |  | 0.1903 | 96.7048 |  |  |  |
|  |  |  |  |  |  |  |  |  |  |  |  |  |
| IC50= |  |  |  |  |  |  |  | IC50= |  |  |  |  |
|  |  |  |  |  |  |  |  |  |  |  |  |  |
|  |  |  |  |  |  |  |  |  |  |  |  |  |
|  | **Blank** | **CC** | **Sample No. Dox/WI38** | | | | | **Sample No.** | | | | |
|  | **1** | **2** | **3** | **4** | **5** | **6** | **7** | **8** | **9** | **10** | **11** | **12** |
| A | B | C | 25uM | 12.5uM | 6.25uM | 3.25uM | 1.55uM |  |  |  |  |  |
| B | B | C | 25uM | 12.5uM | 6.25uM | 3.25uM | 1.55uM |  |  |  |  |  |
| C | B | C | 25uM | 12.5uM | 6.25uM | 3.25uM | 1.55uM |  |  |  |  |  |
| ROBONIK P2000 Eia reader | | | |  |  |  |  |  |  |  |  |  |
| Wave length: 450 nm | | | |  |  |  |  |  |  |  |  |  |
| Reference: 630 nm | | | |  |  |  |  |  |  |  |  |  |
|  | **1** | **2** | **3** | **4** | **5** | **6** | **7** | **8** | **9** | **10** | **11** | **12** |
|  |  |  |  |  |  |  |  |  |  |  |  |  |
| A | 0.001 | 0.486 | 0.166 | 0.227 | 0.311 | 0.354 | 0.397 |  |  |  |  |  |
| B | 0.001 | 0.505 | 0.153 | 0.281 | 0.324 | 0.349 | 0.422 |  |  |  |  |  |
| C | 0.001 | 0.479 | 0.138 | 0.262 | 0.313 | 0.363 | 0.417 |  |  |  |  |  |
| mean | 0.001 | 0.49 | 0.15233 | 0.25667 | 0.316 | 0.35533 | 0.412 | 0 | 0 | 0 | 0 | 0 |
| % viability |  |  | 31.0884 | 52.381 | 64.49 | 72.517 | 84.0816 | 0 | 0 | 0 | 0 | 0 |
| Dox/WI38 | | 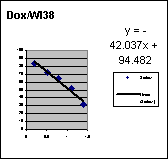 |  |  |  |  |  |  |  | 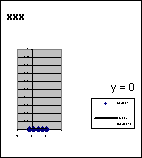 |  |  |
| log conc. | % viability |  |  |  |  |  |  | log conc. | % viability |  |  |  |
| 1.4 | 31.09 |  |  |  |  |  |  | 2 | 0 |  |  |  |
| 1.1 | 52.38 |  |  |  |  |  |  | 1.3979 | 0 |  |  |  |
| 0.8 | 64.49 |  |  |  |  |  |  | 0.7959 | 0 |  |  |  |
| 0.51 | 72.52 |  |  |  |  |  |  | 0.1931 | 0 |  |  |  |
| 0.19 | 84.08 |  |  |  |  |  |  | -0.4089 | 0 |  |  |  |
|  |  |  |  |  |  |  |  |  |  |  |  |  |
| IC50= |  |  |  |  |  |  |  | IC50= |  |  |  |  |
|  |  |  |  |  |  |  |  |  |  |  |  |  |

**Table S7.** **CDK2** inhibitor detailed results.

| **CDK2** |  |  |  |  |  |  |  |  |  |  |  |  |
| --- | --- | --- | --- | --- | --- | --- | --- | --- | --- | --- | --- | --- |
| code | IC50 | conc | log | %inh | T2 | T1 | ∆T | RFU2 | RFU1 | ∆RFU | slope | K.Activity |
| 7e |  | 100 | 2 | 92.7 | 30 | 0 | 30 | 7.26 | 0 | 7.26 | 3.333 | 8.712 |
|  |  | 10 | 1 | 86.1 | 30 | 0 | 30 | 13.88 | 0 | 13.88 | 3.333 | 16.656 |
| 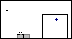  \|  \| \| --- \| |  | 1 | 0 | 68.1 | 30 | 0 | 30 | 31.91 | 0 | 31.91 | 3.333 | 38.292 |
|  |  | 0.1 | -1 | 48.2 | 30 | 0 | 30 | 51.79 | 0 | 51.79 | 3.333 | 62.148 |
|  |  | 0.01 | -2 | 25.8 | 30 | 0 | 30 | 74.15 | 0 | 74.15 | 3.333 | 88.98 |
| EC |  |  |  | 0 | 30 | 0 | 30 | 100 | 0 | 100 | 3.333 | 120 |
|  |  |  |  |  |  |  |  |  |  |  |  |  |
| code | IC50 | conc | log | %inh | T2 | T1 | ∆T | RFU2 | RFU1 | ∆RFU | slope | K.Activity |
| roscovitine |  | 100 | 2 | 90.8 | 30 | 0 | 30 | 9.2 | 0 | 9.23 | 3.333 | 11.234 |
| 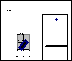  \|  \| \| --- \| |  | 10 | 1 | 85 | 30 | 0 | 30 | 15 | 0 | 15.02 | 3.333 | 16.648 |
|  |  | 1 | 0 | 66.34 | 30 | 0 | 30 | 34.66 | 0 | 34.53 | 3.333 | 35.096 |
|  |  | 0.1 | -1 | 40.8 | 30 | 0 | 30 | 60.2 | 0 | 60.23 | 3.333 | 60.892 |
|  |  | 0.01 | -2 | 25.7 | 30 | 0 | 30 | 74.3 | 0 | 74.3 | 3.333 | 76.006 |
| EC |  |  |  | 0 | 30 | 0 | 30 | 100 | 0 | 100 | 3.333 | 120 |
|  |  |  |  |  |  |  |  |  |  |  |  |  |

|  |  |  |  |
| --- | --- | --- | --- |

**Table S8.** **DHFR** inhibitor Detailed Result.

| DHFR |  |  |  |  |  |  |  |  |  |  |  |  |
| --- | --- | --- | --- | --- | --- | --- | --- | --- | --- | --- | --- | --- |
| code | IC50 | conc | log | %inh | T2 | T1 | ∆T | RFU2 | RFU1 | ∆RFU | slope | K.Activity |
| 8d |  | 100 | 2 | 93 | 30 | 0 | 30 | 6.96 | 0 | 6.96 | 3.333 | 8.352 |
| 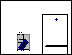  \|  \| \| --- \| |  | 10 | 1 | 85.7 | 30 | 0 | 30 | 14.32 | 0 | 14.32 | 3.333 | 17.184 |
|  |  | 1 | 0 | 67.1 | 30 | 0 | 30 | 32.91 | 0 | 32.91 | 3.333 | 39.492 |
|  |  | 0.1 | -1 | 38.9 | 30 | 0 | 30 | 61.08 | 0 | 61.08 | 3.333 | 73.296 |
|  |  | 0.01 | -2 | 27.5 | 30 | 0 | 30 | 72.54 | 0 | 72.54 | 3.333 | 87.048 |
| EC |  |  |  | 0 | 30 | 0 | 30 | 100 | 0 | 100 | 3.333 | 120 |
|  |  |  |  |  |  |  |  |  |  |  |  |  |
| code | IC50 | conc | log | %inh | T2 | T1 | ∆T | RFU2 | RFU1 | ∆RFU | slope | K.Activity |
| MTX |  | 100 | 2 | 92.7 | 30 | 0 | 30 | 7.28 | 0 | 7.28 | 3.333 | 8.736 |
| 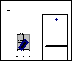  \|  \| \| --- \| |  | 10 | 1 | 87 | 30 | 0 | 30 | 12.96 | 0 | 12.96 | 3.333 | 15.552 |
|  |  | 1 | 0 | 71.4 | 30 | 0 | 30 | 28.55 | 0 | 28.55 | 3.333 | 34.26 |
|  |  | 0.1 | -1 | 43.2 | 30 | 0 | 30 | 56.82 | 0 | 56.82 | 3.333 | 68.184 |
|  |  | 0.01 | -2 | 30.2 | 30 | 0 | 30 | 69.78 | 0 | 69.78 | 3.333 | 83.736 |
| EC |  |  |  | 0 | 30 | 0 | 30 | 100 | 0 | 100 | 3.333 | 120 |
|  |  |  |  |  |  |  |  |  |  |  |  |  |


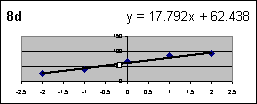

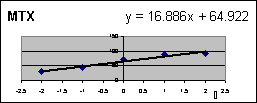


**Table S9**: **Eef2** kinase inhibitor detailed results.

| **ser** | **compound** | | **eEF2**  **IC50** | SD  ± |
| --- | --- | --- | --- | --- |
|  | **code** | **M.W**  **g/mol** | **uM** |  |
| 1 | **8d** | 670 | **0.689** | 0.036 |
| *** | **NH125** | 524.55 | **0.357** | 0.019 |

| **Eef2** |  |  |  |  |  |  |  |  |  |  |  |  |
| --- | --- | --- | --- | --- | --- | --- | --- | --- | --- | --- | --- | --- |
| code | IC50 | conc | log | %inh | T2 | T1 | ∆T | RFU2 | RFU1 | ∆RFU | slope | K.Activity |
| 8d |  | 100 | 2 | 91.7 | 30 | 0 | 30 | 8.31 | 0 | 8.31 | 3.333 | 9.972 |
| 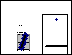 |  | 10 | 1 | 76.5 | 30 | 0 | 30 | 23.51 | 0 | 23.51 | 3.333 | 28.212 |
|  |  | 1 | 0 | 48.2 | 30 | 0 | 30 | 51.82 | 0 | 51.82 | 3.333 | 62.184 |
|  |  | 0.1 | -1 | 32.8 | 30 | 0 | 30 | 67.19 | 0 | 67.19 | 3.333 | 80.628 |
|  |  | 0.01 | -2 | 16.5 | 30 | 0 | 30 | 83.51 | 0 | 83.51 | 3.333 | 100.212 |
| EC |  |  |  | 0 | 30 | 0 | 30 | 100 | 0 | 100 | 3.333 | 120 |
|  |  |  |  |  |  |  |  |  |  |  |  |  |
| code | IC50 | conc | log | %inh | T2 | T1 | ∆T | RFU2 | RFU1 | ∆RFU | slope | K.Activity |
| NH125 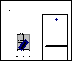 |  | 100 | 2 | 92.6 | 30 | 0 | 30 | 7.42 | 0 | 7.42 | 3.333 | 8.904 |
|  |  | 10 | 1 | 84.3 | 30 | 0 | 30 | 15.72 | 0 | 15.72 | 3.333 | 18.864 |
|  |  | 1 | 0 | 62.7 | 30 | 0 | 30 | 37.29 | 0 | 37.29 | 3.333 | 44.748 |
|  |  | 0.1 | -1 | 33.9 | 30 | 0 | 30 | 66.11 | 0 | 66.11 | 3.333 | 79.332 |
|  |  | 0.01 | -2 | 20.1 | 30 | 0 | 30 | 79.86 | 0 | 79.86 | 3.333 | 95.832 |
| EC |  |  |  | 0 | 30 | 0 | 30 | 100 | 0 | 100 | 3.333 | 120 |
|  |  |  |  |  |  |  |  |  |  |  |  |  |


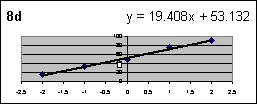

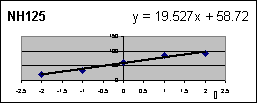


|  |  |  |  |
| --- | --- | --- | --- |
|  |  |  |  |
|  |  |  |  |
|  |  |  |  |
|  |  |  |  |
|  |  |  |  |

**Table S10.** **IKB** inhibitor detailed result.

| **ser** | **compound** | | **IκB**  **IC50** | SD  ± |
| --- | --- | --- | --- | --- |
|  | **code** | **M.W**  **g/mol** | **uM** |  |
| 1 | **8d** | 670 | **0.240** | 0.013 |
| *** | **TPCA-1** | 279.29 | **0.072** | 0.004 |

| **IκB** |  |  |  |  |  |  |  |  |  |  |  |  |
| --- | --- | --- | --- | --- | --- | --- | --- | --- | --- | --- | --- | --- |
| code | IC50 | conc | log | %inh | T2 | T1 | ∆T | RFU2 | RFU1 | ∆RFU | slope | K.Activity |
| 8d |  | 100 | 2 | 93.7 | 30 | 0 | 30 | 6.26 | 0 | 6.26 | 3.333 | 7.512 |
| 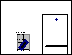  \|  \| \| --- \| |  | 10 | 1 | 86.4 | 30 | 0 | 30 | 13.57 | 0 | 13.57 | 3.333 | 16.284 |
|  |  | 1 | 0 | 63.9 | 30 | 0 | 30 | 36.09 | 0 | 36.09 | 3.333 | 43.308 |
|  |  | 0.1 | -1 | 42.3 | 30 | 0 | 30 | 57.71 | 0 | 57.71 | 3.333 | 69.252 |
|  |  | 0.01 | -2 | 21.8 | 30 | 0 | 30 | 78.19 | 0 | 78.19 | 3.333 | 93.828 |
| EC |  |  |  | 0 | 30 | 0 | 30 | 100 | 0 | 100 | 3.333 | 120 |
|  |  |  |  |  |  |  |  |  |  |  |  |  |
| code | IC50 | conc | log | %inh | T2 | T1 | ∆T | RFU2 | RFU1 | ∆RFU | slope | K.Activity |
| TPCA-1 |  | 100 | 2 | 95.2 | 30 | 0 | 30 | 4.82 | 0 | 4.82 | 3.333 | 5.784 |
| 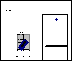  \|  \| \| --- \| |  | 10 | 1 | 88.6 | 30 | 0 | 30 | 11.35 | 0 | 11.35 | 3.333 | 13.62 |
|  |  | 1 | 0 | 71.3 | 30 | 0 | 30 | 28.72 | 0 | 28.72 | 3.333 | 34.464 |
|  |  | 0.1 | -1 | 53.6 | 30 | 0 | 30 | 46.38 | 0 | 46.38 | 3.333 | 55.656 |
|  |  | 0.01 | -2 | 32.5 | 30 | 0 | 30 | 67.52 | 0 | 67.52 | 3.333 | 81.024 |
| EC |  |  |  | 0 | 30 | 0 | 30 | 100 | 0 | 100 | 3.333 | 120 |
|  |  |  |  |  |  |  |  |  |  |  |  |  |


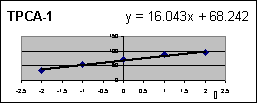

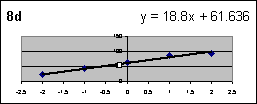


| \|  \| \| --- \| |  |  |  |
| --- | --- | --- | --- | --- |
|  |  |  |  |
|  |  |  |  |
|  |  |  |  |
|  |  |  |  |

**Table S11.** **RET** inhibitor detailed results.

| **ser** | **Compound** | | **RET** | SD  ± |
| --- | --- | --- | --- | --- |
|  | **CODE** | **MW**  **g/mol** | **IC50**  **uM** |  |
| 1 | **7e** | 578 | **0.106** | 0.005 |
| *** | **Staurosporine** | 466.5 | **0.069** | 0.003 |

| **RET** |  |  |  |  |  |  |  |  |  |  |  |  |
| --- | --- | --- | --- | --- | --- | --- | --- | --- | --- | --- | --- | --- |
| code | IC50 | conc | log | %inh | T2 | T1 | ∆T | RFU2 | RFU1 | ∆RFU | slope | K.Activity |
| 7e |  | 100 | 2 | 94.5 | 30 | 0 | 30 | 5.45 | 0 | 5.45 | 3.333 | 6.54 |
|  |  | 10 | 1 | 88.6 | 30 | 0 | 30 | 11.38 | 0 | 11.38 | 3.333 | 13.656 |
| 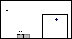  \|  \| \| --- \| |  | 1 | 0 | 72.1 | 30 | 0 | 30 | 27.85 | 0 | 27.85 | 3.333 | 33.42 |
|  |  | 0.1 | -1 | 45.7 | 30 | 0 | 30 | 54.29 | 0 | 54.29 | 3.333 | 65.148 |
|  |  | 0.01 | -2 | 31.5 | 30 | 0 | 30 | 68.53 | 0 | 68.53 | 3.333 | 82.236 |
| EC |  |  |  | 0 | 30 | 0 | 30 | 100 | 0 | 100 | 3.333 | 120 |
|  |  |  |  |  |  |  |  |  |  |  |  |  |
| code | IC50 | conc | log | %inh | T2 | T1 | ∆T | RFU2 | RFU1 | ∆RFU | slope | K.Activity |
| Staurosporine |  | 100 | 2 | 94 | 30 | 0 | 30 | 6.03 | 0 | 6.03 | 3.333 | 7.236 |
| 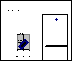  \|  \| \| --- \| |  | 10 | 1 | 88.2 | 30 | 0 | 30 | 11.82 | 0 | 11.82 | 3.333 | 14.184 |
|  |  | 1 | 0 | 74.6 | 30 | 0 | 30 | 25.44 | 0 | 25.44 | 3.333 | 30.528 |
|  |  | 0.1 | -1 | 50.1 | 30 | 0 | 30 | 49.93 | 0 | 49.93 | 3.333 | 59.916 |
|  |  | 0.01 | -2 | 34.5 | 30 | 0 | 30 | 65.48 | 0 | 65.48 | 3.333 | 78.576 |
| EC |  |  |  | 0 | 30 | 0 | 30 | 100 | 0 | 100 | 3.333 | 120 |
|  |  |  |  |  |  |  |  |  |  |  |  |  |


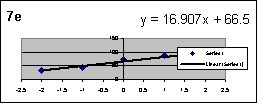

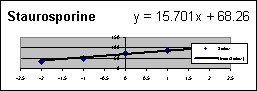

Supplement: Supplementary file 1 — Additional file 1: Fig. S1. FT-IR spectrum of Compound 1. Fig. S2. 1H NMR spectrum of Compound 1. Fig. S3. 13C NMR spectrum of compound 1. Fig. S4. FT-IR spectrum of compound 3. Fig. S5. 1H NMR spectrum of compound 3. Fig. S6. 13C NMR spectrum of compound 3. Fig. S7. FT-IR spectrum of compound 4. Fig. S8. 1H NMR spectrum of compound 4. Fig. S9. 13C NMR spectrum of compound 4. Fig. S10. FT-IR spectrum of compound 5. Fig. S11. 1H NMR spectrum of compound 5. Fig. S12. 13C NMR spectrum of compound 5. Fig. S13. FT-IR spectrum of compound 6. Fig. S14. 1H NMR spectrum of compound 6. Fig. S15. 13C NMR spectrum of compound 6. Fig. S16. FT-IR spectrum of compound 7a. Fig. S17. 1H NMR spectrum of compound 7a. Fig. S18. 13C NMR spectrum of compound 7a. Fig. S19. FT-IR spectrum of compound 7b. Fig. S20. 1H NMR spectrum of compound 7b. Fig. S21. 13C NMR spectrum of compound 7b. Fig. S22. FT-IR spectrum of compound 7c. Fig. S23. 1H NMR spectrum of compound 7c. Fig. S24. 13C NMR spectrum of compound 7c. Fig. S25. FT-IR spectrum of compound 7d. Fig. S26. 1H NMR spectrum of compound 7d. Fig. S27. 13C NMR spectrum of compound 7d. Fig. S28. FT-IR spectrum of compound 7e. Fig. S29. 1H NMR spectrum of compound 7e. Fig. S30. 13C NMR spectrum of compound 7e. Fig. S31. FT-IR spectrum of compound 8a. Fig. S32. 1H NMR spectrum of compound 8a. Fig. S33. 13C NMR spectrum of compound 8a. Fig. S34. FT-IR spectrum of compound 8b. Fig. S35. 1H NMR spectrum of compound 8b. Fig. S36. 13C NMR spectrum of compound 8b. Fig. S37. FT-IR spectrum of compound 8c. Fig. S38. 1H NMR spectrum of compound 8c. Fig. S39. 13C NMR spectrum of compound 8c. Fig. S40. FT-IR spectrum compound 8d. Fig. S41. 1H NMR spectrum compound 8d. Fig. S42. 13C NMR spectrum of compound 8d. Fig. S43. FT-IR spectrum compound 8e. Fig. S44. 1H NMR spectrum compound 8e. Fig. S45. 13C NMR spectrum of compound 8e. Table S1. Raw date of toxicity and viability of compounds 1,3–6 against MCF7. Table S2. Raw date of toxicity and viability of c [file 13065_2024_1139_MOESM1_ESM.docx]
